# Gene mutational pattern and expression level in 560 acute myeloid leukemia patients and their clinical relevance

**DOI:** 10.1186/s12967-017-1279-4

**Published:** 2017-08-22

**Authors:** Yong-Mei Zhu, Pan-Pan Wang, Jin-Yan Huang, Yun-Shuo Chen, Bing Chen, Yu-Jun Dai, Han Yan, Yi Hu, Wen-Yan Cheng, Ting-Ting Ma, Sai-Juan Chen, Yang Shen

**Affiliations:** Department of Hematology, Shanghai Institute of Hematology, RuiJin Hospital Affiliated to Shanghai Jiao Tong University School of Medicine, 197 RuiJin Road II, Shanghai, 200025 China

**Keywords:** Acute myeloid leukemia, Gene expression, Mutation, Prognosis

## Abstract

**Background:**

Cytogenetic aberrations and gene mutations have long been regarded as independent prognostic markers in AML, both of which can lead to misexpression of some key genes related to hematopoiesis. It is believed that the expression level of the key genes is associated with the treatment outcome of AML.

**Methods:**

In this study, we analyzed the clinical features and molecular aberrations of 560 newly diagnosed non-M3 AML patients, including mutational status of *CEBPA*, *NPM1*, *FLT3*, *C*-*KIT*, *NRAS*, *WT1*, *DNMT3A*, *MLL*-*PTD* and *IDH1*/2, as well as expression levels of *MECOM*, *ERG*, *GATA2*, *WT1*, *BAALC*, *MEIS1* and *SPI1*.

**Results:**

Certain gene expression levels were associated with the cytogenetic aberration of the disease, especially for *MECOM*, *MEIS1* and *BAALC*. *FLT3*, *C*-*KIT* and *NRAS* mutations contained conversed expression profile regarding *MEIS1*, *WT1*, *GATA2* and *BAALC* expression, respectively. *FLT3, DNMT3A*, *NPM1* and biallelic *CEBPA* represented the mutations associated with the prognosis of AML in our group. Higher *MECOM* and *MEIS1* gene expression levels showed a significant impact on complete remission (CR) rate, disease free survival (DFS) and overall survival (OS) both in univariate and multivariate analysis, respectively; and an additive effect could be observed. By systematically integrating gene mutational status results and gene expression profile, we could establish a more refined system to precisely subdivide AML patients into distinct prognostic groups.

**Conclusions:**

Gene expression abnormalities contained important biological and clinical informations, and could be integrated into current AML stratification system.

**Electronic supplementary material:**

The online version of this article (doi:10.1186/s12967-017-1279-4) contains supplementary material, which is available to authorized users.

## Background

Acute myeloid leukemia (AML) is a group of hematological malignancies, arising from stem cells, whose leukemogenesis and clinical behavior was deeply affected by the underlying cytogenetic and molecular abnormalities [1–3]. Classic cytogenetic aberrations such as chromosomal translocations to form oncogenic fusion genes via rearrangement of coding sequences of the involved partner genes, such as t(15;17), t(8;21), t(16;16)/inv(16), have long been considered as diagnostic markers of each subgroup of AML, and even served in the surveillance of minimal residual disease (MRD), and more importantly, designing tailored treatment for the disease [4–8]. For more refined stratification, or redefinition of AML, molecular analysis of gene mutation with potential clinical relevance has been more widely used in recent years [9, 10]. Traditionally, it was suggested that genetic abnormalities in leukemia could be roughly grouped into two classes according to their roles in pathogenesis: Class I, mutations involving signal transduction pathways and giving rise to proliferative advantages to leukemia clones, exemplified as *C*-*KIT*, *FLT3* and *NRAS*; and Class II, mutations affecting transcription factors (TF) or co-factors and causing impaired differentiation such as point mutation of *CEBPA*, *AML1* and gene fusion of *AML1*-*ETO* [11]. In our previous work, a Class III mutation associated with epigenetic modifier was proposed, such as *DNMT3A*, *IDH1*, *IDH2*, and *TET2*, which shared a common feature of aggressive diseases, old age and poor prognosis [12]. With the development of second generation sequencing technology, a greater number of new gene mutations were identified in AML; which provides opportunities to more comprehensively understand and overview the gene events in the disease from a panoramic angle. Through whole genome and exome sequencing in 200 AML patients, Ley et al. suggested 9 categories of gene mutations in AML: *NPM1*, activated signaling, myeloid TFs, TF fusions, DNA methylation, chromatin modifier, tumor suppressors, cohesin complex, and spliceosome. However, their function and potential clinical translation in guiding treatment and judging prognosis should be further confirmed in future clinical trials [13].

Until now, the roles of a sizable portion of gene mutations are addressed and integrated in clinical practice. For instance, *FLT3* and *CEBPA* mutations being the most common gene mutations in Western and Chinese populations respectively; represent poor and favorable indicators in AML (biallelic for *CEBPA*) [11, 12, 14–16]. Gene mutations associated with epigenetic modification are also considered as poor factors, such as *MLL*, *DNMT3A*, *TET2*, and *ASXL1* mutations [12, 17, 18], while mutant *NPM1* is regarded as a favorable one [12, 19, 20]. In addition, numerous clinical studies have proven the role of tumor suppressor genes such as *TP53* and *WT1* in cytogenetic normal AML (CN-AML) [21–24] and *C*-*KIT* in core binding factor AML (CBF-AML) [25]. Metallothionein III (MT3) may also act as a tumor suppressor gene of which the promoter hypermethylation can inactivate the gene and downregulate its expression level in pediatric AML [26]. Similar to cytogenetic abnormalities, gene mutation events are now involved in the classification or nomenclature in AML. In a recent large series of 1540 patients, clinical relevance of gene mutations was analyzed, and a new genomic classification of acute myeloid leukemia was proposed, which includes the categories of mutated chromatin, RNA-splicing genes, *TP53* mutations, biallelic *CEBPA* mutations, *MLL* fusion gene, *GATA2*, *MECOM*, *IDH2*, and t(6;9)(p23;q34)/*DEK*-*NUP214*, with each of the subtypes presenting distinct clinical behaviors [27].

How these gene mutations are involved in the leukemogenesis needs further investigation. It is believed that normal hematopoiesis and cellular differentiation is highly dependent on the transcriptional regulation systems. The expression of lineage-determining transcription factor is in strict time order. Gene alterations, including fusions and mutations, could lead to the abnormal expression of key genes, and these kinds of misexpression disrupt the TF-dependent genetic network. In recent years, the expression level of several genes became research interest, exemplified as *MECOM* (also termed *EVI1* and *PRDM3*), *BAALC*, *ERG* and *WT1*. A remote *GATA2* hematopoietic enhancer alteration in inv(3)(q21;q26) by activating *EVI1* expression was reported [28]. It is believed that the expression level of these genes is negatively associated with the treatment outcome of AML [29–33]; however, the results are controversial between western and eastern countries especially for *BAALC* and *ERG* [34, 35]. The role of some new gene markers, such as *MEIS1*, which is up-regulated by *MLL* abnormalities, still needs to be addressed in AML [36, 37].

Hence, we performed this study to systemically investigate the role of a series of gene expression in AML, including previously known ones and newly established ones. Moreover, we intent to integrate these new markers into current established gene mutation profile to provide a more precise stratification of AML.

## Patients and methods

### Patients

The newly diagnosed non-M3 patients were selected from Shanghai Institute of Hematology (SIH). Patients with leukemia either transformed from myelodysplasia syndrome (MDS) or secondary to other malignancies were excluded from this study. Cytogenetic analysis was performed centrally in SIH in every patient. The bone marrow (BM) samples of de novo AML patients were studied mostly by R- and/or G-banding analysis, and were confirmed in most cases with relevant molecular markers [38].

This study was approved by the ethic board of Ruijin hospital. All patients had given informed consent for both treatment and cryopreservation of BM and peripheral blood (PB) according to the Declaration of Helsinki.

### Treatment protocols

Younger AML patients (age ≤ 60) received standard first line treatment of DA like regimen, which consisted of daunorubicin 45–60 mg/m^2^, D1-3; and Ara-C 100 mg/m^2^, D1-7. In the consolidation therapy, they were treated with high-dose cytarabine based chemotherapy for 4 cycles. For old patients (age > 60, n = 86), the treatment was mainly decided by the physician: fit patients underwent a regimen similar to younger patients, but with a reduced consolidation cycles of high dose Ara-C to 2 cycles; unfit patients, underwent either low dose treatment, demethylation treatment or palliative care [39].

### Molecular genetic analysis

Gene mutations/fusions were detected as previously reported [12]. The *WT1* [40] and *ABL1* control gene [41] RQ-PCR assays were performed as described before. The *MECOM*, *ERG*, *GATA2*, *BAALC*, *MEIS1* and *SPI1* expression levels were quantified by using the TaqMan Gene Expression Assay assays, according to the manufacturer’s instructions (Assay ID: Hs00602795_m1, Hs01554629_m1, Hs00231119_m1, Hs00227249_m1, Hs01017441_m1, Hs02786711_m1, respectively). These seven genes’ transcripts were normalized to *ABL1* by using the respective plasmid standards to generate normalized copy numbers. Reactions were performed using ABI ViiA™7 (Life technologies, USA). Each sample was analyzed in duplicate. Data were reported using a common threshold of 0.1. Positive and negative controls were included in all assays.

### Statistical analyses

Fisher’s exact P test was used to compare the gene expression levels in different subgroups, as well as the difference of CR rates. One way Anova test was used to compare the clinical features such as age and WBC count in different groups. OS was measured from the date of disease diagnosis to death (failure) or alive at last follow-up (censored). DFS was defined as the duration from the documentation of CR to treatment failure such as relapse, refractory disease, death, or alive in CR at last follow-up (censored). Kaplan–Meier analysis was used to calculate the distribution of OS and DFS. Hazard ratio analysis was performed to compare the difference of survivals. Binary logistic regression and COX model was used for the multivariate analysis of associations between mutational status and the achievement of CR and OS and DFS, respectively. A limited backward selection procedure was used to exclude redundant variates. All above statistical procedures were performed with the SPSS statistical software package, version 16.0.

## Results

### Gene expression level in AML

Gene expression levels of *MECOM*, *ERG*, *WT1*, *GATA2*, *BAALC*, *MEIS1* and *SPI1* in bone marrow (BM) of de novo AML patients were shown in Additional file 1: Figure S1.

We cut the expression level of *WT1*, *BAALC* and *ERG* into high and low group by their median values in patients according to the previous reports [30, 32]. For the continuance of analysis, the cut-off levels of *MECOM*, *MEIS1*, *GATA2* and *SPI1* were also chosen at their median values. Survival analysis showed that median value as cut-off value could separate patients with different prognosis in *MECOM*, *MEISI* and *SPI1* expression group (OS: P < 0.001, P < 0.001 and P = 0.010, respectively), while using cut-off value at quartile 1 (Q1) or Q3 would lose the power to separate the patients (Additional file 2: Figure S2).

### Patient characteristics and gene expression level

560 patients were entered into this study including 474 young patients (age ≤ 60 years old) and 86 elderly patients (age > 60 years old). The patients were classified into 3 groups: Group 1, Core Binding Factor AML (CBF-AML), which includes 89 patients, Group 2, Cytogenetic intermediate risk AML, which includes 401 patients with normal karyotype (CN-AML, 320 patients) or cytogenetic aberration without prognostic significance, and Group 3, Cytogenetic high risk patients (55 patients) group. Among group 2, young patients with normal karyotype (265 patients) or insignificant cytogenetic aberration accounted for 84% (336 patients), while elderly patients accounted for 16% (65 patients) including 55 CN-AML patients. Cytogenetic results were failed or unavailable in 15 patients. The distribution of the patients in FAB classification and cytogenetic abnormalities of enrolled patients was listed in Additional file 3: Table S1.

Age was strongly associated with several gene expression levels. The patients with higher *MEISI* expression had the higher median age (P = 0.026). Similarly, in old patients (age > 60), more patients harbored higher *MEIS1* and *SPI1* expression levels (66.3% vs. 33.7%, P = 0.001; 60.5% vs. 39.5%, P = 0.035, respectively). Female patients tended to have higher expression of *MECOM*, *MEIS1* and *WT1* (P = 0.038, P = 0.010 and P = 0.032, respectively). The high *SPI1* expression group manifested the feature of high WBC count at disease presenting (P = 0.003). The detailed clinical features were shown in Table 1.

**Table 1:** Clinical characteristics and gene expression level

Gene expression	Age (years)			Gender, n (%)		WBC count, ×10^9^/L
	Median (range)	≤60, n (%)	>60, n (%)	Male	Female	Median (range)
*MECOM* (missing = 12)						
Low (n = 274)	42.5 (1–81)	238 (51.4)	36 (42.4)	168 (61.3)	106 (38.7)	14.2 (0.34–453.0)
High (n = 274)	43 (1–83)	225 (48.6)	49 (57.6)	144 (52.6)	130 (47.4)	13.9 (0.50–376.8)
P	0.448		0.125	0.038		0.515
*MEIS1*						
Low (n = 280)	42 (2–80)	251 (53.0)	29 (33.7)	175 (62.5)	105 (37.5)	13.7 (0.65–453.0)
High (n = 280)	45 (1–83)	223 (47.0)	57 (66.3)	145 (51.8)	135 (48.2)	15.3 (0.34–389.4)
P	0.026		0.001	0.010		0.409
*SPI1*						
Low (n = 280)	42 (1–81)	246 (51.9)	34 (39.5)	165 (58.9)	115 (41.1)	10.6 (0.5–453.0)
High (n = 280)	44 (1–83)	228 (48.1)	52 (60.5)	155 (55.4)	125 (44.6)	16.9 (0.34–298.5)
P	0.113		0.035	0.393		0.003
*ERG*						
Low (n = 280)	44 (1–81)	238 (50.2)	42 (48.8)	151 (53.9)	129 (46.1)	13.1 (0.34–389.4)
High (n = 280)	41 (1–83)	236 (49.8)	44 (51.2)	169 (60.4)	111 (39.6)	15.8 (0.50–453.0)
P	0.490		0.815	0.124		0.210
*WT1* (missing = 2)						
Low (n = 279)	43 (1–83)	237 (50.2)	42 (48.8)	172 (61.6)	107 (38.4)	13.5 (0.34–376.8)
High (n = 279)	43 (2–81)	235 (49.8)	44 (51.2)	147 (52.7)	132 (47.3)	16.9 (0.50–453.0)
P	0.873		0.815	0.032		0.406
*GATA2*						
Low (n = 280)	43 (2–83)	239 (50.4)	41 (47.7)	162 (57.9)	118 (42.1)	11.0 (0.34–376.8)
High (n = 280)	43 (1–81)	235 (49.6)	45 (52.3)	158 (56.4)	122 (43.6)	17.5 (0.50–453.0)
P	0.832		0.639	0.733		0.003
*BAALC*						
Low (n = 280)	45 (1–79)	239 (50.4)	41 (47.7)	153 (54.6)	127 (45.4)	14.2 (0.34–389.4)
High (n = 280)	41 (1–83)	235 (49.6)	45 (52.3)	167 (59.6)	113 (40.4)	14.1 (0.6–453.0)
P	0.279		0.639	0.232		0.878

*WBC* white blood cell, *n* number

### Cytogenetic aberrations and gene expression level

**Fig. 1:**
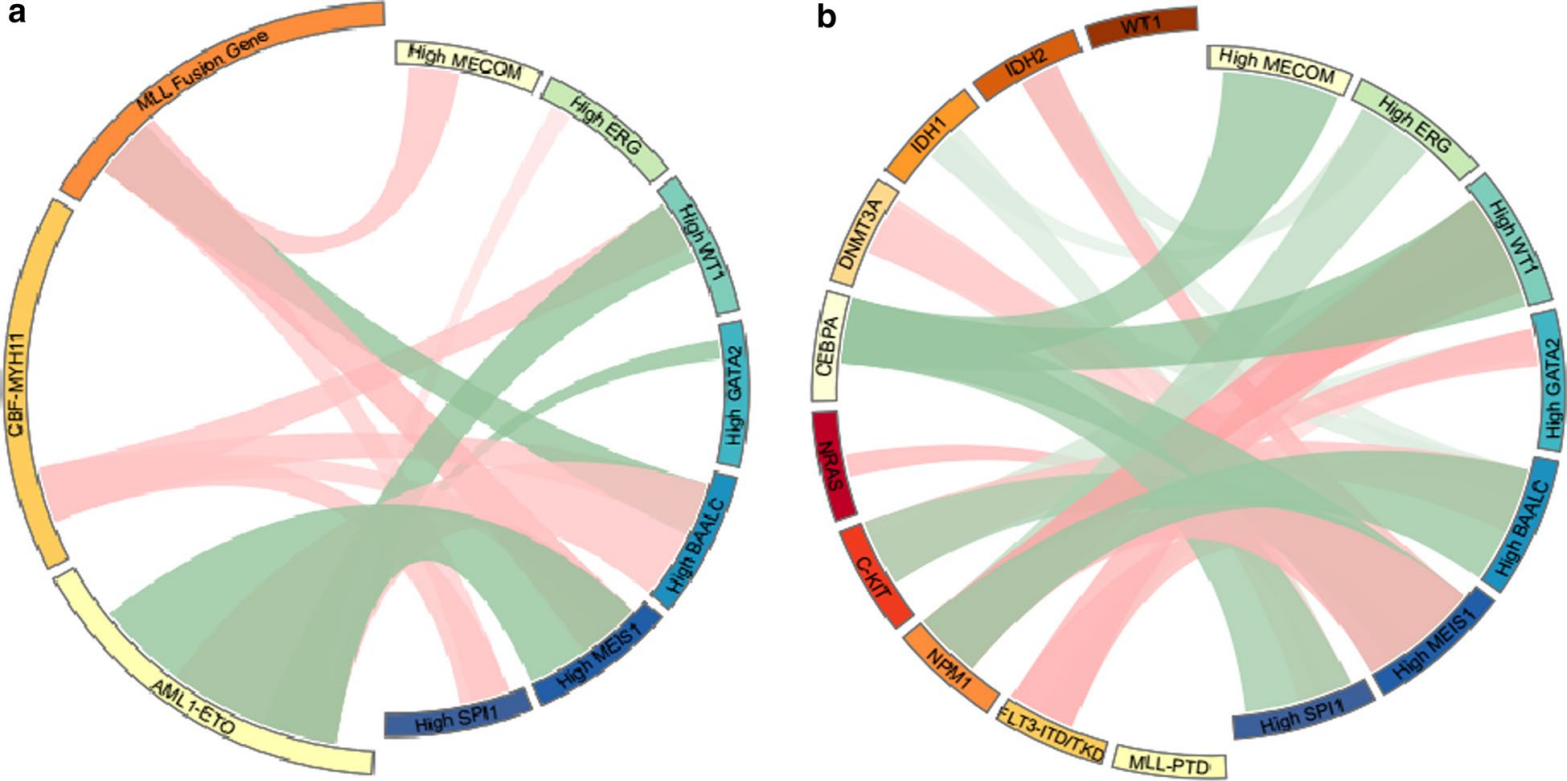
Circos figure of mutual relationship between gene mutational status and investigated gene expression level. *Green line* indicated the negative relationship while the *red line* indicated positive relationship with statistical significance. **a** Relationship of gene fusions with gene expression level. **b** Relationship of gene mutations with gene expression level. The detailed numeric data were seen in Table 2 and Additional file 5: Table S3

There was a difference of gene expression levels including those of *MECOM*, *MEIS1*, *SPI1*, *ERG*, *WT1*, *GATA2*, and *BAALC* in different cytogenetic risk groups. In CBF-AML, *MEIS1* and *WT1* (both P < 0.001) were lower in CBFα-AML (t(8;21)), but higher in CBFβ-AML (inv(16)) (P = 0.001 and <0.001, respectively); while *BAALC* was higher in both groups (both P < 0.001). And *SPI1* was high in CBFβ-AML (P < 0.001) as previously reported [42]. In CN-AML, *MECOM* and *BAALC* were lower (both P < 0.001), while *GATA2* was higher (P = 0.014). Particularly, *MECOM* tended to have a higher level in poor risk factors both in 11q23 rearrangement (P = 0.001) and others (P = 0.015). 11q23 abnormalities were associated with higher expressions of *MECOM* (P = 0.001), *MEIS1* (P = 0.003), *SPI1* (P = 0.031), *WT1* (P = 0.032), and interestingly, a lower *BAALC* expression (P = 0.003) in our group. Figure 1 and Table 2 showed the relationship of cytogenetic abnormalities and gene expression level.

**Table 2:** Cytogenetic characteristics and gene expression level

Gene expression	Cytogenetic characteristics, n (%) (failed = 15)					
	CBF-AML		Cytogenetic intermediate-risk		Cytogenetic high-risk	
	t(8;21)	inv(16)	Normal cytogenetics	Others	11q23	Others
*MECOM*						P < 0.001
Low	34 (12.5)	9 (3.3)	181 (66.8)	34 (12.5)	5 (1.8)	8 (3.0)
High	40 (15.3)	6 (2.3)	130 (49.6)	44 (16.8)	22 (8.4)	20 (7.6)
P	0.364	0.472	<0.001	0.165	0.001	0.015
*MEIS1*						P < 0.001
Low	72 (26.6)	1 (0.4)	152 (56.1)	30 (11.1)	6 (2.2)	10 (3.7)
High	2 (0.7)	14 (5.1)	168 (61.3)	51 (18.6)	21 (7.7)	18 (6.6)
P	<0.001	0.001	0.215	0.013	0.003	0.128
*SPI1*						P < 0.001
Low	34 (12.5)	0 (0.0)	172 (63.2)	46 (16.9)	8 (2.9)	12 (4.4)
High	40 (14.7)	15 (5.5)	148 (54.2)	35 (12.8)	19 (7.0)	16 (5.9)
P	0.463	<0.001	0.032	0.179	0.031	0.444
*ERG*						P = 0.004
Low	28 (10.4)	5 (1.9)	160 (59.5)	54 (20.1)	11 (4.1)	11 (4.1)
High	46 (16.7)	10 (3.6)	160 (58.0)	27 (9.8)	16 (5.8)	17 (6.2)
P	0.033	0.208	0.721	0.001	0.358	0.274
*WT1*						P < 0.001
Low	55 (20.4)	0 (0.0)	152 (56.3)	41 (15.2)	8 (3.0)	14 (5.2)
High	19 (7.0)	15 (5.5)	166 (60.8)	40 (14.7)	19 (7.0)	14 (5.1)
P	<0.001	<0.001	0.286	0.862	0.032	0.976
*GATA2*						P = 0.082
Low	46 (17.0)	10 (3.7)	145 (53.5)	44 (16.2)	12 (4.4)	14 (5.2)
High	28 (10.2)	5 (1.8)	175 (63.9)	37 (13.5)	15 (5.5)	14 (5.1)
P	0.021	0.183	0.014	0.370	0.574	0.976
*BAALC*						P < 0.001
Low	9 (3.3)	0 (0.0)	180 (66.7)	48 (17.8)	21 (7.8)	12 (4.4)
High	65 (23.6)	15 (5.5)	140 (50.9)	33 (12.0)	6 (2.2)	16 (5.8)
P	<0.001	<0.001	<0.001	0.058	0.003	0.468

### Gene mutational status and gene expression level

Out of 560 AML patients, 116 (20.7%) patients were with *FLT3*-ITD/TKD mutations, 45 (8.0%) were with *NRAS* mutations, 59 (10.5%) were with *C*-*KIT* mutations, 100 (17.9%) were with *NPM1* mutations, 38 (6.8%) were with *WT1* mutations, 118 (21.1%) were with *CEBPA* mutations, 62 (11.1%) were with *DNMT3A* mutations, 79 (14.1%) were with *IDH1/2* mutations, and 27 (4.8%) were with *MLL*-PTD mutations, respectively. The distribution of gene mutations was accordant to previous reports. The detailed distribution of gene mutations in different cytogenetic risk group was shown in Additional file 4: Table S2.

The potential relationship of mutual co-existence and co-exclusion was observed between gene mutation group and investigated gene expression level. *FLT3* mutations were associated with high *MEIS1* (P < 0.001), *WT1* (P < 0.001), and *GATA2* (P = 0.004) and lower *BAALC* (P = 0.007) expressions, respectively; while *C*-*KIT* was associated with lower *MEIS1* (P < 0.001), *WT1* (P < 0.001) and *GATA2* (P = 0.008) and higher *BAALC* (P < 0.001) expressions, respectively. At the same time, another class I mutation, *NRAS* was not associated with any gene expression level. The mutual exclusion of Class II mutations was also reflected in gene expression level. *CEBPA* was associated with lower *MECOM* (P < 0.001), *MEISI* (P < 0.001), *SPI1* (P < 0.001), and *WT1* (P < 0.001) expressions, respectively. *NPM1* was related with lower *ERG* (P < 0.001) and *BAALC* (P < 0.001), but higher *MEIS1* (P < 0.001), *WT1* (P < 0.001) and *GATA2* (P = 0.001) expression levels, respectively. Other potential mutual relationship with gene expression level was also observed in epigenetic modifier gene mutations, such as *DNMT3A* and *IDH1/2*, respectively. The detailed data were shown in Fig. 1 and Additional file 5: Table S3. Similarly, the relationship of gene mutational status and gene expression level in intermediate risk AML was shown in Additional file 6: Table S4.

### Treatment outcome

#### Response to induction therapy

Firstly, we validated the prognostic value of known gene mutations in cytogenetic intermediate risk patients of our group and also in a separate group of young patients who received uniformed treatment. In univariate analysis, it was shown that *FLT3* (P = 0.036) and biallelic *CEBPA* (P = 0.009) mutations were associated with lower and higher CR rate, respectively. In different gene expression groups, higher *MECOM*, *MEIS1* and *SPI1* expression was associated with a lower CR rate (58.0% vs. 74.4%, P = 0.001, 61.2% vs. 75.8%, P = 0.002, and 62.3% vs. 72.5%, P = 0.030, respectively), while other factors were not associated with the induction outcome of the cytogenetic intermediate risk patients (Table 3).

**Table 3:** CR rate of different gene mutation and expression group in intermediate risk group

Gene mutation	CR no (%)	Gene expression	CR no (%)
*FLT3* ITD/TKD		*MECOM*	
Mutated	60 (59.4)	Low	160 (74.4)
Not mutated	212 (70.7)	High	101 (58.0)
P	0.036	P	0.001
*NRAS*		*MEIS1*	
Mutated	25 (78.1)	Low	138 (75.8)
Not mutated	247 (66.9)	High	134 (61.2)
P	0.194	P	0.002
*C*-*KIT* (NA = 2)		*SPI1*	
Mutated	19 (79.2)	Low	158 (72.5)
Not mutated	271 (67.9)	High	114 (62.3)
P	0.223	P	0.030
*DNMT3A*		*ERG*	
Mutated	33 (61.1)	Low	151 (70.6)
Not mutated	239 (68.9)	High	121 (64.7)
P	0.256	P	0.211
*IDH1* (NA = 1)		*WT1*	
Mutated	23 (60.5)	Low	136 (70.5)
Not mutated	249 (68.6)	High	136 (66.0)
P	0.311	P	0.341
*IDH2*		*GATA2*	
Mutated	22 (62.9)	Low	128 (67.7)
Not mutated	250 (68.3)	High	144 (67.9)
P	0.510	P	0.966
*NPM1*/*DNMT3A*		*BAALC*	
*NPM1*-mut/*DNMT3A*-wt	48 (78.7)	Low	159 (69.7)
Others	224 (65.9)	High	113 (65.3)
P	0.049	P	0.348
*WT1*			
Mutated	26 (76.5)		
Not mutated	245 (66.9)		
P	0.255		
*CEBPA*			
Biallelic mutated	59 (83.1)		
Monoallelic mutated	21 (61.8)		
Not mutated	189 (64.7)		
P	0.009		
*MLL*-*PTD*			
Mutated	14 (60.9)		
Not mutated	258 (68.3)		
P	0.462		

A complete list of covariates that entered multivariate model was indicated in Table 4. Multivariate analysis indicated that age and cytogenetic risk remained independent prognostic factors for CR induction outcome, while molecular profile added more informative value in predicting the treating results, exemplified as *NPM1*-mut/*DNMT3A*-wt (OR = 3.389, 95% CI 1.519–7.562; P = 0.003) and high *MECOM* and *MEIS1* expression (OR = 0.576, 95% CI 0.377–0.880; P = 0.011, and OR = 0.389, 95% CI 0.251–0.603, P < 0.001, respectively), respectively. A multivariate analysis was also performed in cytogenetic intermediate risk group AML patients. The results were similar in terms of gene expression level (Additional file 7: Table S5). And similar results of both univariate analysis and multivariate analysis could be observed in younger patients as well (Additional file 8: Table S6, Additional file 9: Table S7, Additional file 10: Table S8).

**Table 4:** Multivariate analysis of prognostic value of AML

Variables	CR		OS		DFS	
	OR (95% CI)	P	HR (95% CI)	P	HR (95% CI)	P
Age	0.968 (0.957–0.980)	<0.001	1.023 (1.016–1.030)	<0.001	1.018 (1.008–1.028)	<0.001
WBC		NS		NS		NS
Cytogenetic risk	0.894 (0.800–0.998)	0.046	1.093 (1.032–1.158)	0.002	1.106 (1.019–1.201)	0.016
*FLT3*-ITD/TKD		NS	1.408 (1.074–1.845)	0.013	1.492 (1.010–2.203)	0.044
Biallelic *CEBPA*		NS	0.524 (0.325–0.845)	0.008		NS
NPM1-mut/DNMT3A-wt	3.389 (1.519–7.562)	0.003	0.523 (0.355–0.770)	0.001		NS
*MLL*-PTD		NS		NS		NS
*DNMT3A* mutation		NS		NS		NS
High *MECOM*	0.576 (0.377–0.880)	0.011	1.642 (1.296–2.081)	<0.001	1.788 (1.319–2.424)	<0.001
High *MESI1*	0.389 (0.251–0.603)	<0.001	1.432 (1.114–1.840)	0.005		NS
High *SPI1*		NS		NS		NS

*NS* no significance

#### Survival analysis

Survival stratification was performed in the intermediate cytogenetic group, which consisted major part of AML (nearly 70%). In our group, the median OS and DFS were observed at 17 ± 2.3 and 27.5 ± 4.2 months (24 ± 3.1 and 30 ± 5.0 months for young patients), respectively (among which, the median OS and DFS of CN-AML were 19 ± 2.9 and 30 ± 4.8 months (26 ± 4.8 and 34 ± 6.9 months for young patients), respectively).

Firstly, survival analysis was performed according to the gene mutational status. In univariate analysis, biallelic *CEBPA* mutations were associated with favorable OS (HR = 0.329, P < 0.001) and DFS (HR = 0.691, P = 0.002), respectively, as well as *NPM1*-mut/*DNMT3A*-wt (OS: HR = 2.009, P = 0.010; DFS: HR = 2.039, P = 0.044), which was accordant to previous study. In contrast, *FLT3*-ITD/TKD and *DNMT3A* mutations were associated with poor prognosis (Additional file 11: Figure S3). Statistical significance was not observed in other gene mutation groups.

**Fig. 2:**
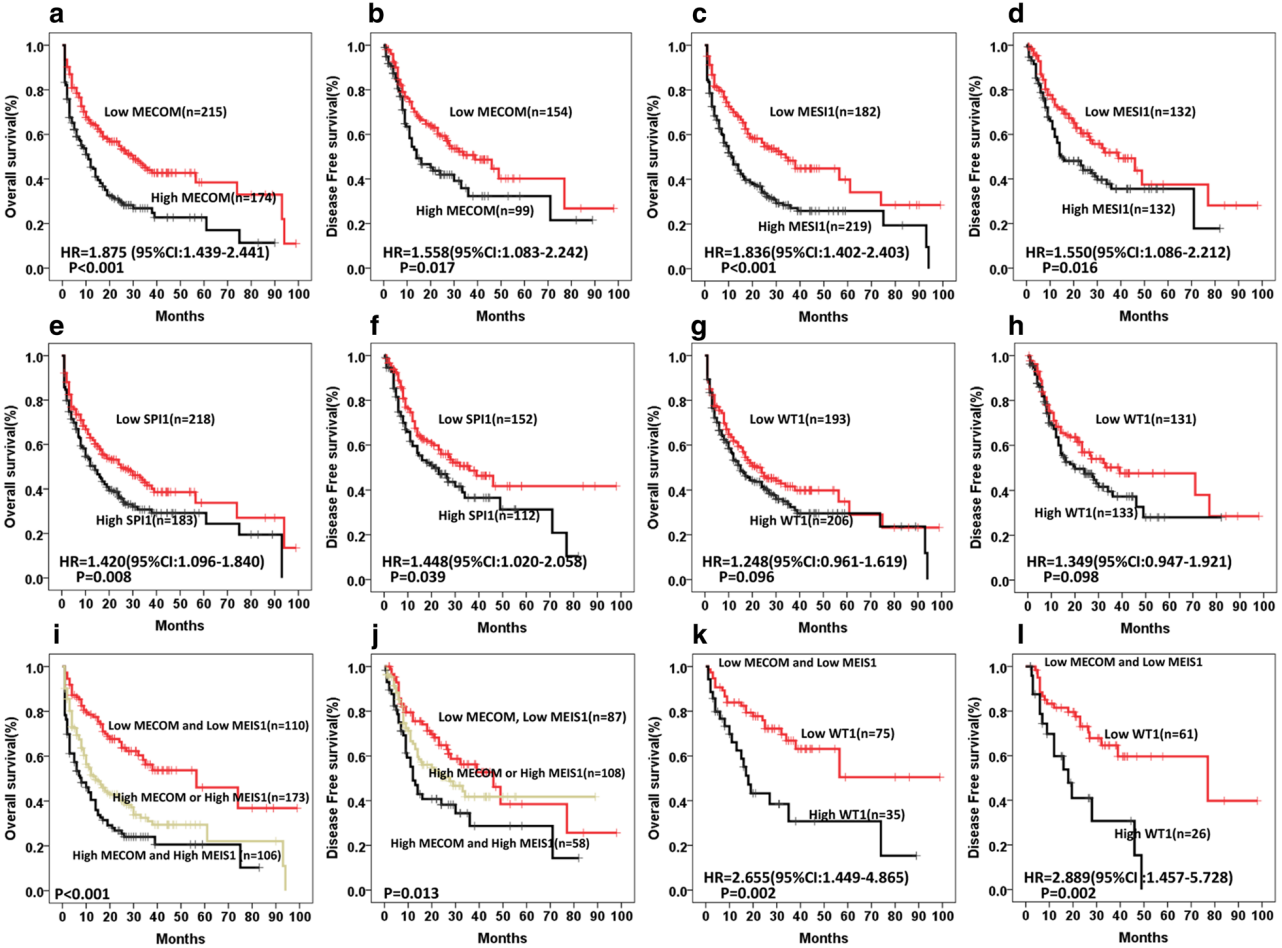
Comparison of OS and DFS between patients with low or high gene expression with statistical significance in univariate analysis. **a**, **b** The median OS and DFS of patients with low or high *MECOM* expression were 30 ± 4.7 months versus 11 ± 1.8 months (*P* < 0.001), and 39 ± 8.1 months versus 14 ± 3.8 months (*P* = 0.017), respectively.** c**, **d** The median OS and DFS of patients with low or high *MEIS1* expression were 34 ± 5.3 months versus 11.5 ± 1.7 months (*P* < *0.001*), and 39 ± 8.7 months versus 14.5 ± 4.1 months (*P* = 0.016), respectively. **e**, **f** The median OS and DFS of patients with low or high *SPI1* expression were 25 ± 5.4 months versus 14 ± 2.1 months (*P* = 0.008), and 36 ± 7.7 months versus 21 ± 5.3 months (*P* = 0.039), respectively. **g**, **h** The median OS and DFS of patients with low or high *WT1* expression were 22 ± 4.2 months versus 15 ± 2.3 months (*P* = 0.096) and 39 ± 14.4 months versus 20 ± 5.9 months (*P* = 0.098), respectively. **i**, **j** Patients with low expression of both *MECOM* and *MEIS1* were compared to those with either or both high expression of *MECOM* and *MEIS1*. The median OS were 56.5 ± 17.5, 14 ± 2.6 and 8 ± 2.2 months (*P* < 0.001). The median DFS were 46 ± 8.2, 27.5 ± 7.9 and 12 ± 1.7 months (*P* = *0.013*). The hazard ratios (HR) of high *MECOM* or *MEIS1* for OS and DFS were 2.076 (95% CI 1.455–2.962) and 1.379 (95% CI 0.893–2.131), respectively, while the HR of High *MECOM* and High *MEIS1* were 3.040 (95% CI 2.088–4.427) and 2.024 (95% CI 1.265–3.238), respectively. **k**, **l** The OS and DFS were compared in a subgroup of patients with low expression of both *MECOM* and *MEIS1.* The median OS and DFS of patients with low or high *WT1* expression were NR versus 18 ± 2.4 months (*P* = 0.002), and 77 ± 32.7 months versus 18 ± 4.5 months (*P* = 0.002), respectively

Similarly, according to gene expression level, in univariate analysis, high *MECOM* (HR = 1.875, P < 0.001 for OS; and HR = 1.558, P = 0.017 for DFS), high *MEIS1* (HR = 1.836, P < 0.001 for OS; and HR = 1.550, P = 0.016 for DFS) and high *SPI1* (HR = 1.402, P = 0.008 for OS; and HR = 1.448, P = 0.039 for DFS) were associated with the survival. The two parameters, *MECOM* and *MEIS1*, even had an additive effect, high *MECOM* and high *MEIS1* group showed worst prognosis, low *MECOM* and low *MEIS1* group presented most favorable treatment outcome, while either high *MECOM* or *MEIS1* group was in the middle (Fig. 2i, j). *WT1* could be served in further discriminating the patients with both low *MECOM* and *MEIS1* group, the high *WT1* expression was associated with poor OS (HR = 2.655, P = 0.002) and DFS (HR = 2.889, P = 0.002).

**Fig. 3:**
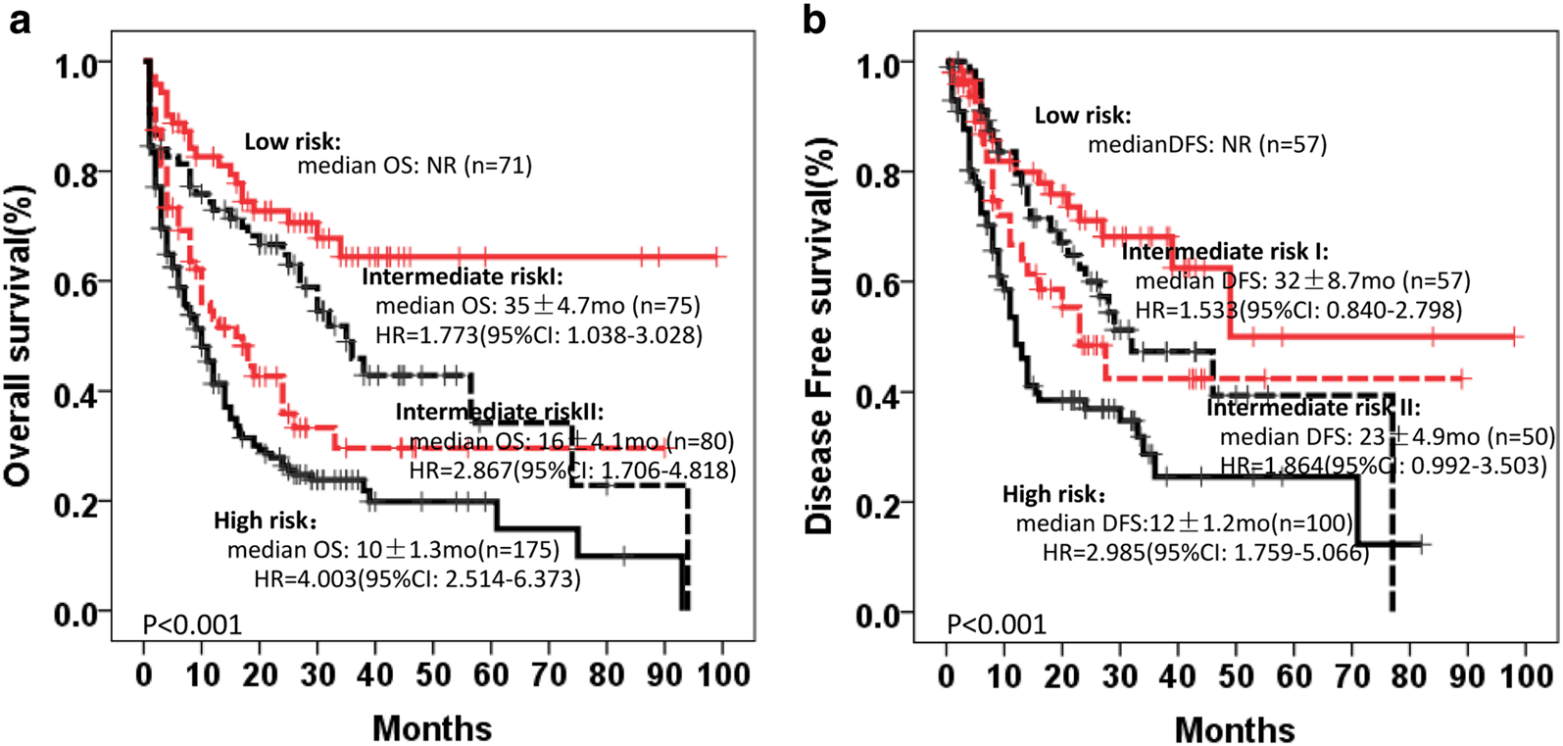
Risk stratification of AML according to gene mutations and gene expression level. Low risk: biallelic *CEBPA* mutation; Intermediate risk I: low *MECOM* and *MEIS1* without biallelic *CEBPA* mutation or *NPM1*-mut/*DNMT3A*-wt; Intermediate risk II: others; High risk: *FLT3*-ITD/TKD with the absence of *NPM1*-mut/*DNMT3A*-wt or *DNMT3A* mutation or high *MECOM* and *MEIS1*. **a** OS, **b** DFS

According to above analysis and inter-relationship of gene expression level, we further stratified cytogenetic intermediate risk intermediate AML patients into 4 groups: (1) low risk: biallelic *CEBPA* mutations; (2) intermediate risk I: low *MECOM* and *MEIS1* expression without biallelic *CEBPA* mutations or *NPM1*-mut/*DNMT3A*-wt; (3) intermediate risk II: others; (4) high risk: *FLT3*-ITD/TKD with the absence of *NPM1*-mut/*DNMT3A*-wt or *DNMT3A* mutations or high *MECOM* and *MEIS1* expression (Fig. 3).

In multivariate analysis, in whole group of AML patients, age and cytogenetic risk remained independent prognostic factors. In terms of molecular factors, *FLT3* mutations (for both OS and DFS), biallelic *CEBPA* mutations (for OS), *NPM1*-mut/*DNMT3A*-wt (for OS), as well as high *MECOM* (both for OS and DFS) and high *MEIS1* (for OS) expressions were independent prognostic factors for AML (Table 4). And in intermediate risk group, similar results were also achieved (Additional file 7: Table S5). For young patients, all the above survival analysis were performed and demonstrated with the similar results (Additional file 9: Table S7, Additional file 10: Table S8; Additional file 12: Figure S4, Additional file 13: Figure S5, Additional file 14: Figure S6).

## Discussion

Cytogenetic analysis serves as a traditional tool to discriminate the prognosis of the AML [10, 43–46]. However, the shortcomings such as lack of sensitivity, labor and time cost limits the further application of this technique. Genetic mutations that escape cytogenetic detection have increasingly been discovered and these mutations may serve as potential markers to extend the prognostic parameters in AML. Numerous systemic investigations involving a series of genes have been performed in AML using first or second generation of sequencing techniques, and prognostic value has been analyzed, especially for the most common ones, such as *NPM1*, *CEBPA*, *FLT3* etc. [11, 13, 47]. New generation sequencing broadens our eyes to view more deeply about these gene mutations, not only the clinical behavior and prognosis, but also the disease nature. More classes of gene mutations were named, and even used in defining the special subgroup of AML [27]. A significant progress could be observed that the diagnosis of the disease strides from a simple morphological 7 FAB subtypes to a much complicated system involving cytogenetic aberrations, gene mutations, and even gene expression levels. Nowadays, examination of gene mutations was almost routinely performed all over the world in AML field, and integrated into the daily practice in treating AML.

However, in addition to gene mutations, some gene expression levels, which are caused by the regulation of a certain gene, or even several genes, are also involved in leukemogenesis. Among them, *MECOM* expression was the most widely reported. It was firstly reported to be associated with a specific translocation with extreme poor prognosis, inv(3)(q21q26.2), which has lower incidence in AML [48, 49]. Then, it was also identified to have a high expression level in AML with other cytogenetic abnormalities and even CN-AML, exemplified as in *MLL*-AF9 pediatric AML, which was associated with poor prognosis [31]; as well as in M4/5, or *MLL* rearrangement in another Japanese series with 130 pediatric AML patients [35]. In addition to *MECOM*, several groups use *WT1* expression level to monitor the minimal residual diseases (MRD) in BM and PB in AML, whose increasing strongly indicates poor prognosis and relapse [40]. When compared with universal recognition, *MECOM* and *WT1* over-expression are poor indicators, while clinical value of *BAALC* and *ERG* are controversial. Some genes of myeloid transcriptional factors are also drawn of attraction, such as *GATA2* and *SPI1*; especially for *MEIS1*, which is proved to be regulated by *MLL* mutation in previous reports [36]. Although great efforts have been made in recent decades, systemic examination of gene expression level and their cytogenetic and gene mutation background in AML is still lack. We performed this study to examine the cytogenetic abnormalities, gene mutational profile in 560 AML patients, and more importantly, a series of gene expression levels, such as *MECOM*, *WT1*, *ERG*, *BAALC*, and 3 new ones with potential value, *GATA2*, *SPI1* and *MEIS1*, to try to address this question.

Firstly, we identified that certain gene expression levels were associated with the cytogenetic aberrations of the disease. In this study, *MECOM* expression was identified to be low in CN-AML group, but high in 11q23 aberration group. *MEIS1* expression was low in t(8;21) group, but high in the group with poor cytogenetic makers. High *BAALC* expression was associated with CBF-AML, but less distributed in CN- and 11q23 AML. Moreover, gene mutational status was also associated with the gene expression level. *FLT3* was associated with a high *MEIS1*, *WT1*, and *GATA2* and lower *BAALC* expression, respectively; while *C*-*KIT* was associated with a lower *MEIS1*, lower *WT1* and *GATA2* and higher *BAALC* expression, respectively. And interestingly, *NRAS* was not associated with any gene expression level. Above results seemed to be mutually exclusive. *CEBPA*, as the most common mutation type in Chinese population, seemed to have favorable gene expression profile: lower *MECOM* (P < 0.001), *MEISI* (P < 0.001), *SPI1* (P < 0.001), and *WT1* (P < 0.001) expression, respectively. Mutant *NPM1* was related with lower *ERG* (P < 0.001), *BAALC* (P < 0.001), but higher *MEIS1* (P < 0.001), *WT1* (P < 0.001) and *GATA2* (P = 0.001), respectively. As we know, *FLT3*, *C*-*KIT*, *CEBPA* and *NPM1* mutations were considered to have prognostic value in predicting the prognosis of AML, and their related gene expression profile might be the reason. We believe that certain mutations elicit numerous expressional changes in other genes which are associated with leukemogenesis, leading to different clinical behaviors of the diseases. Such kind of mutual relationship with gene expression level was also observed in epigenetic modifier gene mutations.

In treatment outcome analysis, univariate analysis showed that *FLT3* and higher *MECOM*, *MEIS1* and *SPI1* expressions were associated with a lower CR rate (P = 0.036, 0.001, 0.002 and 0.030 respectively), while *CEBPA* (P = 0.009) mutations were associated with a higher CR rate. Higher *MECOM* and *MEIS1* expressions remained significant in multivariate analysis, while *NPM1*-mut/*DNMT3A*-wt appeared to be an independent factor (P = 0.003). In survival, biallelic *CEBPA* mutations and *NPM1*-mut/*DNMT3A*-wt were associated with favorable OS (P < 0.001 and P = 0.010, respectively) and DFS (P = 0.002 and P = 0.044, respectively), respective, while *FLT3*-ITD/TKD (P < 0.001 and P < 0.001, respectively) and *DNMT3A* (P = 0.014 and P = 0.023, respectively) were associated with poor prognosis. As for gene expression profile, high *MECOM* (P < 0.001 for OS; and P = 0.017 for DFS, respectively) and high *MEIS1* (P < 0.001 for OS; and P = 0.016 for DFS, respectively) levels were associated with the survival. An additive effect could be observed when we combined the two gene expression levels together, high *MECOM* and high *MEIS1* group showed worst prognosis, low *MECOM* and low *MEIS1* group presented most favorable treatment outcome. Furthermore, *WT1* could help to separate the low risk group of low *MECOM* and *MEIS1* group into a more refined subgroup, the high *WT1*/low *MECOM* and *MEIS1* expression was associated with relative poor OS (HR = 2.655, P = 0.002) and DFS (HR = 2.889, P = 0.002).

Finally, we have established a new system to stratify the AML integrating cytogenetic risk, gene mutational status and gene expression profile. Through examination of traditional cytogenetic markers, gene mutations, exemplified as *DNMT3A*, *MLL*, *NPM1*, *CEBPA, FLT3* mutations etc., and more importantly gene expression profile, especially for *MECOM*, *MEIS1* and *WT1*, one could discriminate the AML patients with different clinical behaviors.

## Conclusions

Gene expression aberrations are associated with the cytogenetic abnormalities and gene mutations in AML, as well as the clinical behavior of the patients. Of note, their value in predicting the prognosis of AML was demonstrated in this study.

## Additional files

**Additional file 1: Figure S1.** Gene expression of *MECOM*, *ERG*, *WT1*, *GATA2*, *BAALC*, *MEIS1* and *SPI1* in bone marrow (BM) of de novo AML patients was normalized to ABL expression. Each median value after a log-transformation is indicated by a horizontal line.

**Additional file 2: Figure S2.** Kaplan–Meier survival analysis for overall survival (OS) using different cut-offs of relative gene expression. Log-rank P value was portrayed for each survival analysis.

**Additional file 3: Table S1.** FAB subgroups and cytogenetic abnormalities in AML patients.

**Additional file 4: Table S2.** The distribution of gene mutations in different cytogenetic risk groups.

**Additional file 5: Table S3.** The relationship of gene mutational status and gene expression level.

**Additional file 6: Table S4.** The relationship of gene mutational status and gene expression level (intermediate risk).

**Additional file 7: Table S5.** Multivariate analysis of intermediate risk group.

**Additional file 8: Table S6.** CR rate of different gene mutation and expression groups in intermediate risk group (young AML patients).

**Additional file 9: Table S7.** Multivariate analysis of prognostic value of young AML patients.

**Additional file 10: Table S8.** Multivariate analysis of intermediate risk group (young AML patients).

**Additional file 11: Figure S3.** Kaplan–Meier curves for OS and DFS according to genotypes.

**Additional file 12: Figure S4.** Kaplan–Meier curves of young AML patients for OS and DFS according to genotypes.

**Additional file 13: Figure S5.** Comparison of OS and DFS between young AML patients with low or high gene expression with statistical significance in univariate analysis.

**Additional file 14: Figure S6.** Risk stratification of young AML according to gene mutations and gene expression levels.

## Abbreviations

AML: acute myeloid leukemia
MRD: minimal residual disease
TF: transcription factors
CN-AML: cytogenetic normal AML
CBF-AML: core binding factor AML
SIH: Shanghai Institute of Hematology
MDS: myelodysplasia syndrome
BM: bone marrow
PB: peripheral blood
OS: overall survival
DFS: disease free survival
