# Supplementary material for: Gene mutational pattern and expression level in 560 acute myeloid leukemia patients and their clinical relevance
Source: J Transl Med. 2017 Aug 22;15:178. doi: 10.1186/s12967-017-1279-4 (PMC5568401; doi:10.1186/s12967-017-1279-4)

**Figure S1.** Gene expression of *MECOM*, *ERG, WT1*, *GATA2*, *BAALC*, *MEIS1* and *SPI1* in bone marrow(BM) of *de novo* AML patients was normalized to *ABL* expression. Each median value after a log-transformation is indicated by a horizontal line.


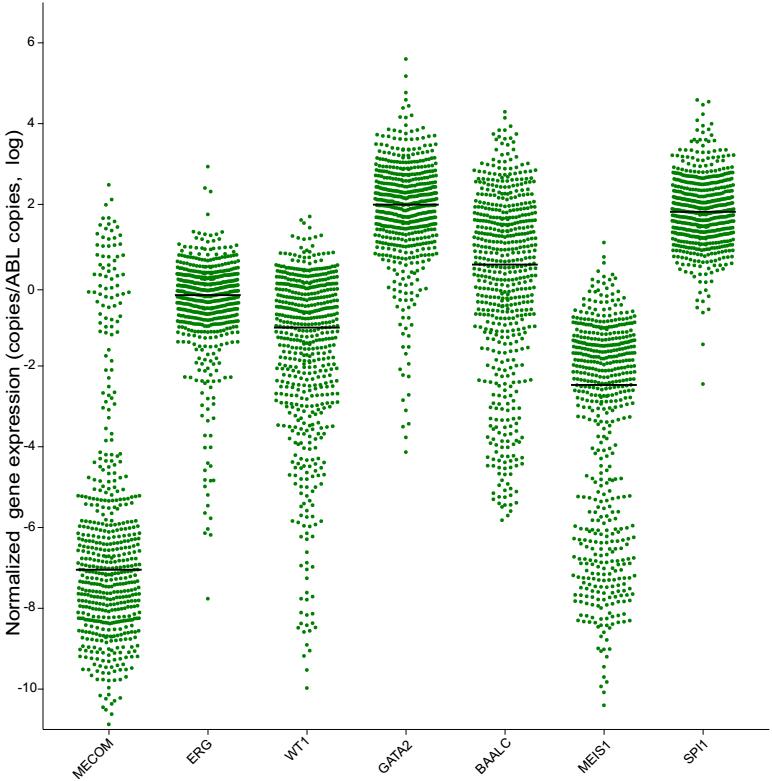

Supplement: Supplementary file 1 — Additional file 1: Figure S1. Gene expression of MECOM, ERG, WT1, GATA2, BAALC, MEIS1 and SPI1 in bone marrow (BM) of de novo AML patients was normalized to ABL expression. Each median value after a log-transformation is indicated by a horizontal line. [file 12967_2017_1279_MOESM1_ESM.docx]
