# Supplementary material for: Gene mutational pattern and expression level in 560 acute myeloid leukemia patients and their clinical relevance
Source: J Transl Med. 2017 Aug 22;15:178. doi: 10.1186/s12967-017-1279-4 (PMC5568401; doi:10.1186/s12967-017-1279-4)
Supplement: Supplementary file 2 — Additional file 2: Figure S2. Kaplan–Meier survival analysis for overall survival (OS) using different cut-offs of relative gene expression. Log-rank P value was portrayed for each survival analysis. [file 12967_2017_1279_MOESM2_ESM.docx]

**Figure S2.** Kaplan Meier survival analysis for overall survival (OS) using different cut-offs of relative gene expression. Log-rank P value was portrayed for each survival analysis. (A) Median as the cut-off; (B) Q3 as the cut-off, (C) Q1 as the cut-off

**
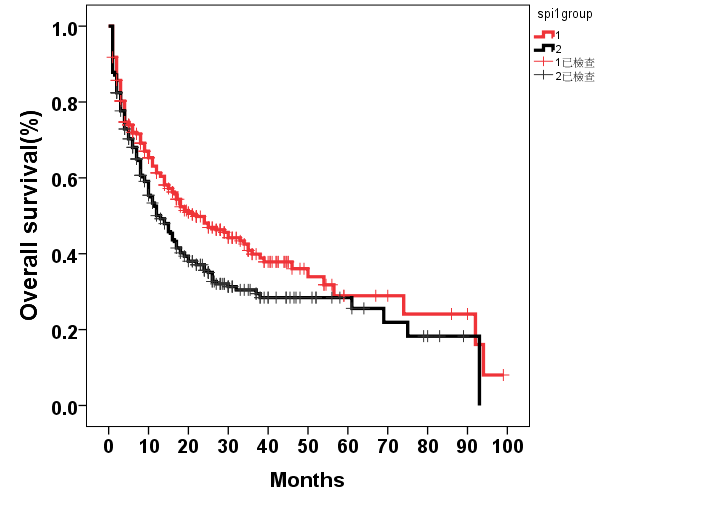

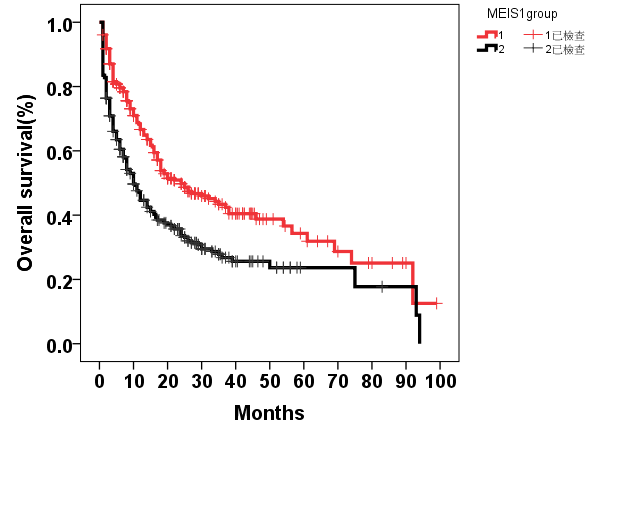

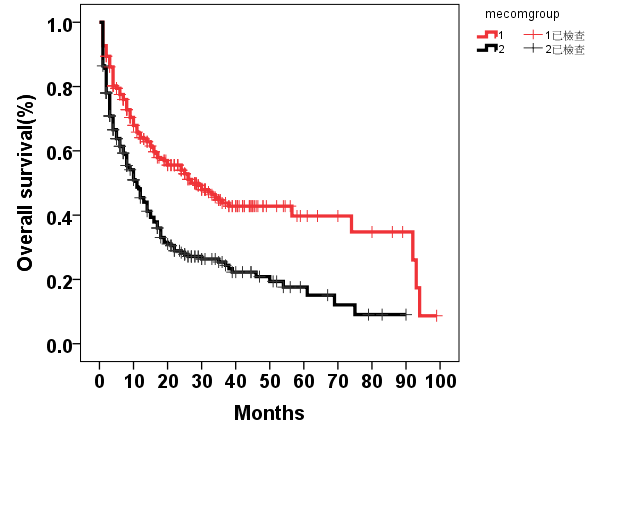
A**

SPI1

MEIS1

MECOM

P=0.010

P<0.001

P<0.001


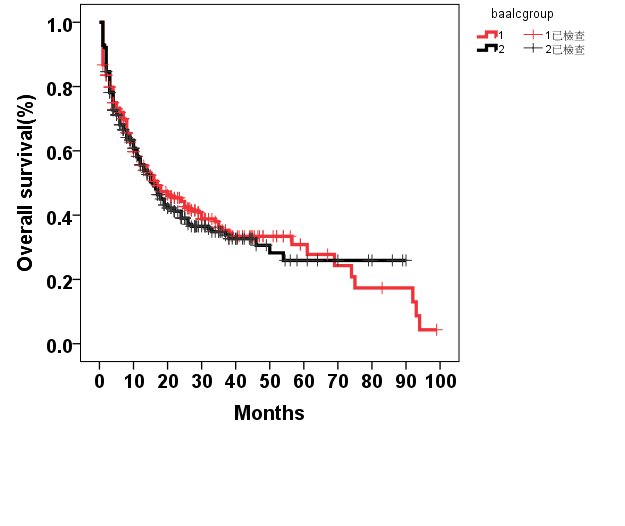

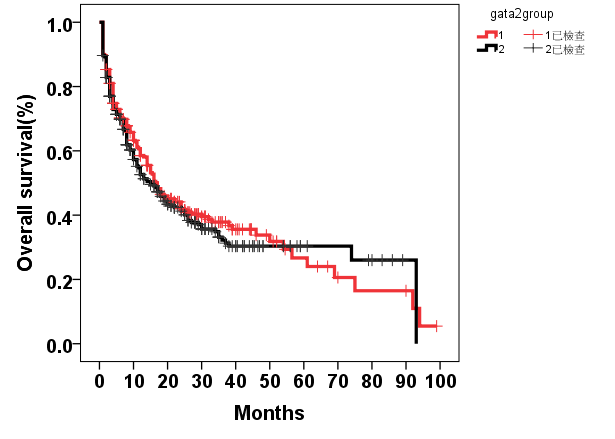

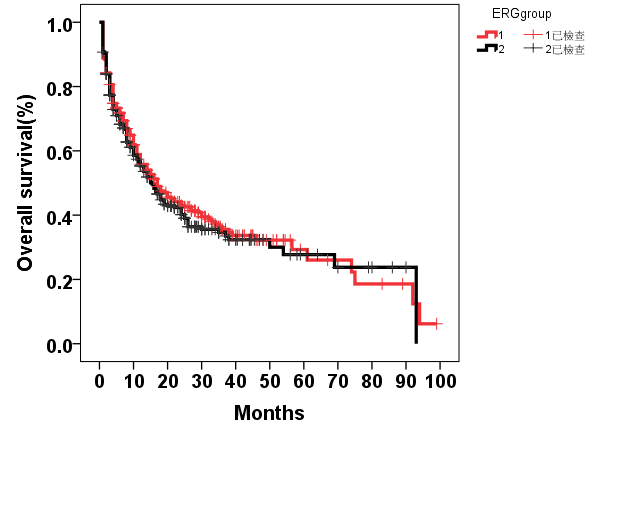

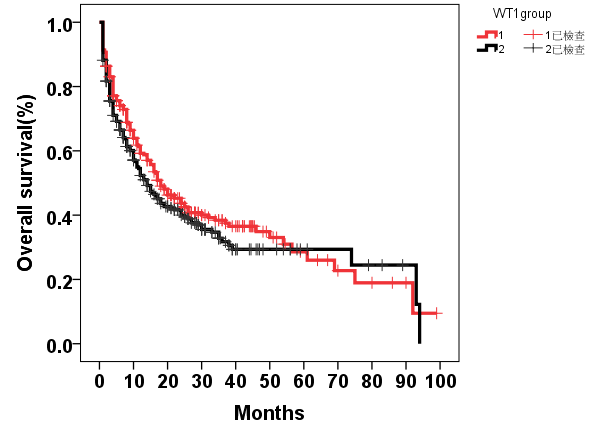


BAALC

ERG

WT1

GATA2

P=0.672

P=0.496

P=0.529

P=0.169

**B
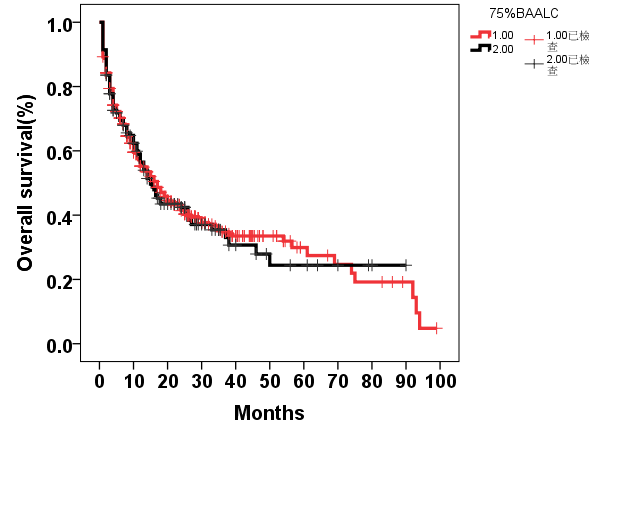

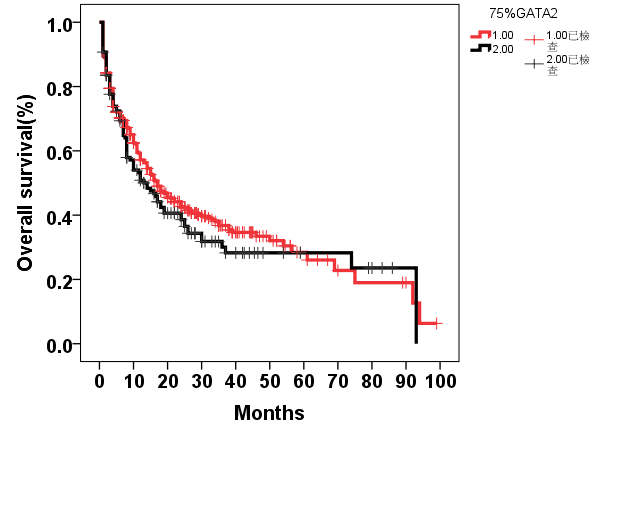

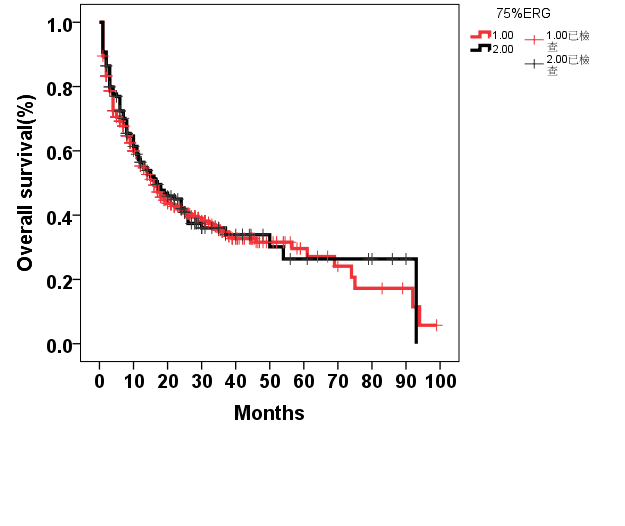

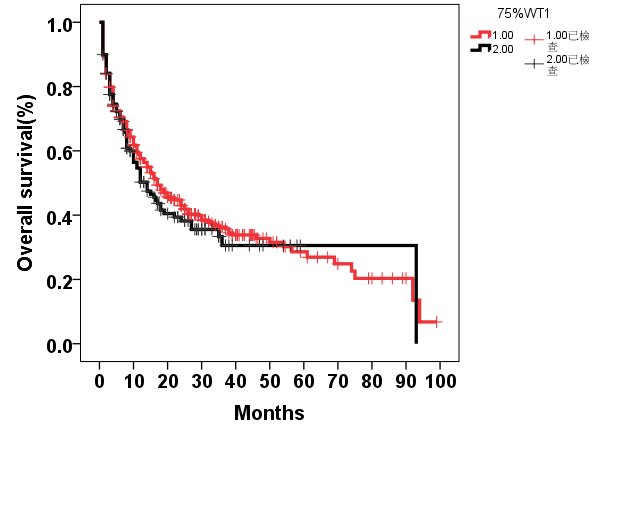
**

WT1

BAALC

GATA2

ERG

P=0.862

P=0.291

P=0.768

P=0.424

**
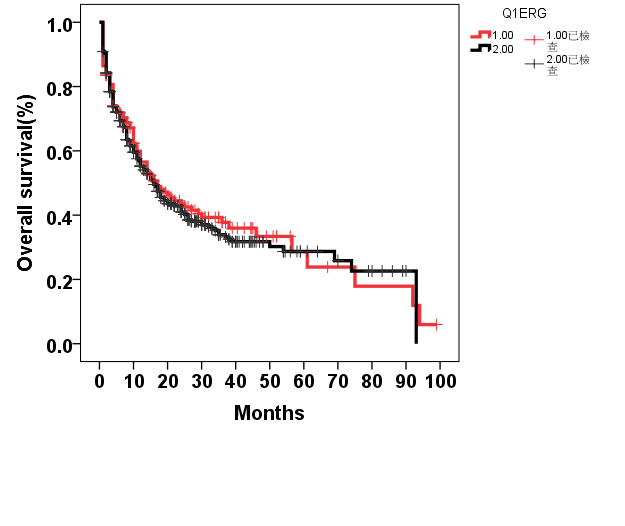
C**
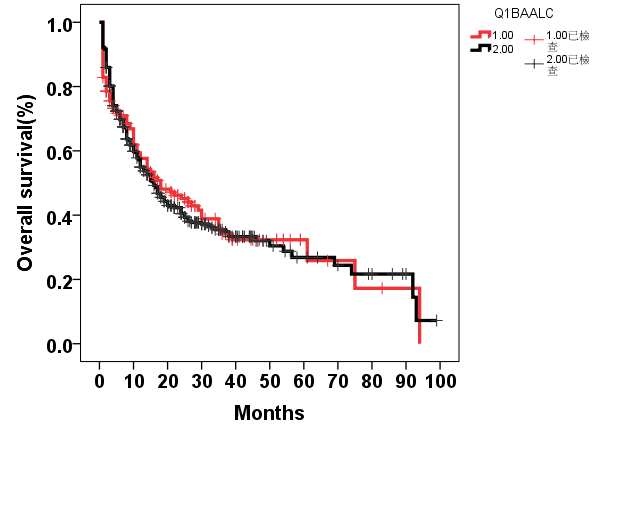

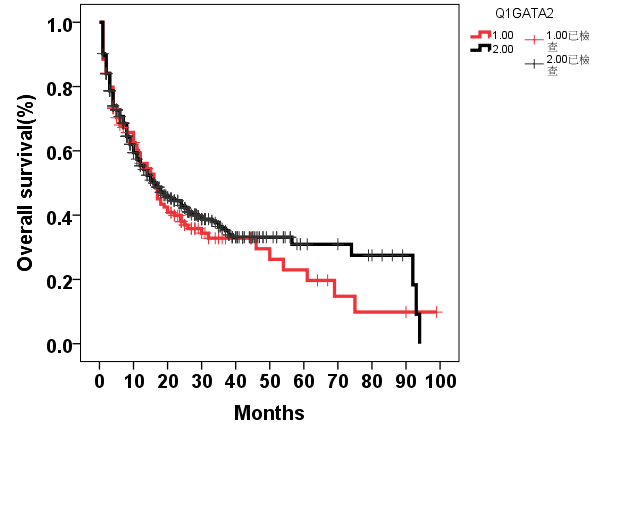

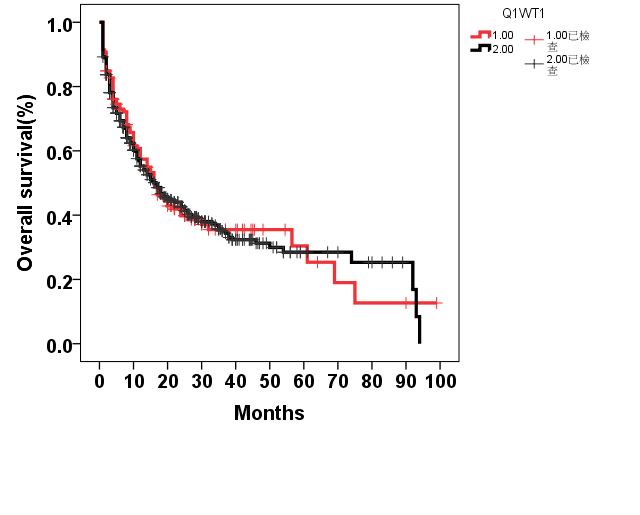


BAALC

GATA2

ERG

WT1

P=0.788

P=0.45

P=0.616

P=0.838
