# Supplementary material for: Gene mutational pattern and expression level in 560 acute myeloid leukemia patients and their clinical relevance
Source: J Transl Med. 2017 Aug 22;15:178. doi: 10.1186/s12967-017-1279-4 (PMC5568401; doi:10.1186/s12967-017-1279-4)
Supplement: Supplementary file 3 — Additional file 3: Table S1. FAB subgroups and cytogenetic abnormalities in AML patients. [file 12967_2017_1279_MOESM3_ESM.docx]

**Table S1.** FAB subgroups and cytogenetic abnormalities in AML patients

| **FAB subtype.** | **No(%)** | **Cytogenetics** | **No(%)** |
| --- | --- | --- | --- |
| **M0** | 0(0.00%) | **CBF-AML** | 89(15.89%) |
| **M1** | 21(3.75%) | **t(8;21)** | 74(13.21%) |
| **M2** | 119(21.25%) | **Inv(16)** | 15(2.68%) |
| **M3** | 0(0.00%) | **Intermediate-risk** | 401(71.6%) |
| **M4** | 220(39.29%) | **Normal cytogenetics** | 320(57.1%) |
| **M5** | 141(25.18%) | **Other** | 81(14.5%) |
| **M6** | 16(2.86%) | **Poor-risk** | 55(9.8%) |
| **M7** | 4(0.71%) | **11q23 rearrangement** | 27(4.8%) |
| **Not classified** | 39(6.96%) | **Other** | 28(5.0%) |
|  |  | **Not classified** | 15(2.7%) |
