# Supplementary material for: Gene mutational pattern and expression level in 560 acute myeloid leukemia patients and their clinical relevance
Source: J Transl Med. 2017 Aug 22;15:178. doi: 10.1186/s12967-017-1279-4 (PMC5568401; doi:10.1186/s12967-017-1279-4)
Supplement: Supplementary file 4 — Additional file 4: Table S2. The distribution of gene mutations in different cytogenetic risk groups. [file 12967_2017_1279_MOESM4_ESM.docx]

**Table S2.** The distribution of gene mutations in different cytogenetic risk groups

| **Gene Mutation** | **Cytogenetic characteristics, n (%)** | | | | | | | | |
| --- | --- | --- | --- | --- | --- | --- | --- | --- | --- |
|  | **CBF-AML** | | **Cytogenetic Intermediate-risk** | | | **Cytogenetic**  **High-risk** | | **Miss** | **Total** |
|  | **t(8;21)**  (n=74) | **Inv(16)**  (n=15) | **Normal**  (n=320) | | **Others**  (n=81) | **11q23**  (n=27) | **Others**  (n=28) | **n=15** | **n=560** |
| ***FLT3 ITD/TKD*** | 3(4.1) | 1(6.7) | | 85(26.6) | 16(19.8) | 5(18.5) | 3(10.7) | 3(20.0) | 116(20.7) |
| ***NRAS*** | 3(4.1) | 2(13.3) | | 24(7.5) | 8(9.9) | 3(11.1) | 3(10.7) | 2(13.3) | 45(8.0) |
| ***C-KIT*** | 32(43.2) | 2(13.3) | | 21(6.6) | 3(3.7) | 0(0.0) | 1(3.6) | 0(0.0) | 59(10.5) |
| ***NPM1*** | 3(4.1) | 0(0.0) | | 82(25.6) | 11(13.6) | 0(0.0) | 0(0.0) | 4(26.7) | 100(17.9) |
| ***WT1*** | 3(4.1) | 0(0.0) | | 28(8.8) | 6(7.4) | 0(0.0) | 1(3.6) | 0(0.0) | 38(6.8) |
| ***CEBPA*** | 5(6.8) | 0(0.0) | | 94(29.4) | 12(14.8) | 2(7.4) | 2(7.1) | 3(20.0) | 118(21.1) |
| ***DNMT3A*** | 0(0.0) | 0(0.0) | | 46(14.4) | 8(9.9) | 2(7.4) | 3(10.7) | 3(20.0) | 62(11.1) |
| ***IDH1*** | 2(2.7) | 0(0.0) | | 32(10.0) | 6(7.4) | 0(0.0) | 1(3.6) | 1(6.7) | 42(7.5) |
| ***IDH2*** | 1(1.4) | 0(0.0) | | 31(9.7) | 4(4.9) | 0(0.0) | 0(0.0) | 1(6.7) | 37(6.6) |
| ***MLL-*PTD** | 0(0.0) | (0.0) | | 22(6.9) | 1(1.2) | 2(7.4) | 2(7.1) | 0(0.0) | 27(4.8) |
