# Supplementary material for: Gene mutational pattern and expression level in 560 acute myeloid leukemia patients and their clinical relevance
Source: J Transl Med. 2017 Aug 22;15:178. doi: 10.1186/s12967-017-1279-4 (PMC5568401; doi:10.1186/s12967-017-1279-4)
Supplement: Supplementary file 5 — Additional file 5: Table S3. The relationship of gene mutational status and gene expression level. [file 12967_2017_1279_MOESM5_ESM.docx]

**Table S3.** The relationship of gene mutational status and gene expression level

| **Gene Expression** | **Gene mutations, n (%)** | | | | | | | | | |
| --- | --- | --- | --- | --- | --- | --- | --- | --- | --- | --- |
|  | ***FLT3* ITD/TKD** | ***NRAS*** | ***C-KIT***  **(NA=2)** | ***NPM1*** | ***CEBPA***  **(NA=4 )** | ***WT1***  **(NA=2)** | ***DNMT3A*** | ***IDH1***  **(NA=1)** | ***IDH2*** | ***MLL-PTD*** |
| ***MECOM*(missing=12)** | |  |  |  |  |  |  |  |  |  |
| Low (n=274) | 61(22.3) | 22(8.0) | 29(10.6) | 55(20.1) | 88(32.1) | 24(8.8) | 27(9.9) | 22(8.0) | 22(8.0) | 13(4.7) |
| High (n=274) | 51(18.6) | 22(8.0) | 29(10.6) | 43(15.7) | 28(10.3) | 14(5.1) | 33(12.0) | 20(7.3) | 15(5.5) | 13(4.7) |
| **P** | 0.289 | 1.000 | 0.988 | 0.181 | <0.001 | 0.093 | 0.412 | 0.757 | 0.233 | 1.000 |
| ***MEIS1*** |  |  |  |  |  |  |  |  |  |  |
| Low (n=280) | 35(12.5) | 24(8.6) | 50(17.9) | 7(2.5) | 103(37.1) | 19(6.8) | 17(6.1) | 20(7.2) | 9(3.2) | 12(4.3) |
| High (n=280) | 81(28.9) | 21(7.5) | 9(3.2) | 93(33.2) | 15(5.4) | 19(6.8) | 45(16.1) | 22(7.9) | 28(10.0) | 15(5.4) |
| **P** | <0.001 | 0.641 | <0.001 | <0.001 | <0.001 | 0.982 | <0.001 | 0.757 | 0.001 | 0.554 |
| ***SPI1*** |  |  |  |  |  |  |  |  |  |  |
| Low (n=280) | 52(18.6) | 15(5.4) | 26(9.3) | 53(18.9) | 87(31.4) | 23(8.2) | 16(5.7) | 31(11.1) | 24(8.6) | 9(3.2) |
| High (n=280) | 64(22.9) | 30(10.7) | 33(11.8) | 47(16.8) | 31(11.1) | 15(5.4) | 46(16.4) | 11(3.9) | 13(4.6) | 18(6.4) |
| **P** | 0.211 | 0.020 | 0.335 | 0.508 | <0.001 | 0.179 | <0.001 | 0.001 | 0.061 | 0.076 |
| ***ERG*** |  |  |  |  |  |  |  |  |  |  |
| Low (n=280) | 52(18.6) | 25(8.9) | 23(8.2) | 72(25.7) | 61(21.9) | 18(6.4) | 33(11.8) | 29(10.4) | 28(10.0) | 10(3.6) |
| High (n=280) | 64(22.9) | 20(7.1) | 36(12.9) | 28(10) | 57(20.6) | 20(7.2) | 29(10.4) | 13(4.7) | 9(3.2) | 17(6.1) |
| **P** | 0.211 | 0.437 | 0.073 | <0.001 | 0.711 | 0.720 | 0.590 | 0.011 | 0.001 | 0.167 |
| ***WT1*(missing=2)** | |  |  |  |  |  |  |  |  |  |
| Low (n=279) | 33(11.8) | 23(8.2) | 43(15.5) | 24(8.6) | 89(32.1) | 16(5.8) | 28(10.0) | 20(7.2) | 14(5.0) | 9(3.2) |
| High (n=279) | 82(29.4) | 22(7.9) | 16(5.8) | 76(27.2) | 28(10.1) | 22(7.9) | 34(12.2) | 22(7.9) | 23(8.2) | 18(6.5) |
| **P** | <0.001 | 0.876 | <0.001 | <0.001 | <0,001 | 0.324 | 0.419 | 0.757 | 0.126 | 0.076 |
| ***GATA2*** |  |  |  |  |  |  |  |  |  |  |
| Low (n=280) | 44(15.7) | 26(9.3) | 39(14.0) | 35(12.5) | 57(20.5) | 17(6.1) | 30(10.7) | 21(7.5) | 22(7.9) | 15(5.4) |
| High (n=280) | 72(25.7) | 19(6.8) | 20(7.1) | 65(23.2) | 61(21.9) | 21(7.5) | 32(11.4) | 21(7.5) | 15(5.4) | 12(4.3) |
| **P** | 0.004 | 0.277 | 0.008 | 0.001 | 0.678 | 0.516 | 0.788 | 0.990 | 0.234 | 0.554 |
| ***BAALC*** |  |  |  |  |  |  |  |  |  |  |
| Low (n=280) | 71(25.4) | 21(7.5) | 13(4.7) | 94(33.6) | 52(18.7) | 17(6.1) | 42(15.0) | 22(7.9) | 27(9.6) | 16(5.7) |
| High (n=280) | 45(16.1) | 24(8.6) | 46(16.5) | 6(2.1) | 66(23.7) | 21(7.6) | 20(7.1) | 20(7.2) | 10(3.6) | 11(3.9) |
| **P** | 0.007 | 0.641 | <0.001 | <0.001 | 0.146 | 0.487 | 0.003 | 0.757 | 0.004 | 0.324 |
