# Supplementary material for: Gene mutational pattern and expression level in 560 acute myeloid leukemia patients and their clinical relevance
Source: J Transl Med. 2017 Aug 22;15:178. doi: 10.1186/s12967-017-1279-4 (PMC5568401; doi:10.1186/s12967-017-1279-4)
Supplement: Supplementary file 6 — Additional file 6: Table S4. The relationship of gene mutational status and gene expression level (intermediate risk). [file 12967_2017_1279_MOESM6_ESM.docx]

**Table S4.** The relationship of gene mutational status and gene expression level(intermediate risk)

| **Gene Expression** | **Gene mutations, n (%)** | | | | | | | | | |
| --- | --- | --- | --- | --- | --- | --- | --- | --- | --- | --- |
|  | ***FLT3* ITD/TKD** | ***NRAS*** | ***C-KIT***  **(NA=2)** | ***NPM1*** | ***CEBPA***  **(NA=4 )** | ***WT1***  **(NA=1)** | ***DNMT3A*** | ***IDH1***  **(NA=1)** | ***IDH2*** | ***MLL-PTD*** |
| ***MECOM*(missing=12)** | |  |  |  |  |  |  |  |  |  |
| Low (n=215) | 55(25.6) | 18(8.4) | 15(7.0) | 53(24.7) | 83(38.6) | 21(9.8) | 25(11.6) | 20(9.3) | 21(9.8) | 12(5.6) |
| High (n=174) | 42(24.1) | 13(7.5) | 8(4.6) | 38(21.8) | 21(12.2) | 13(7.5) | 27(15.5) | 18(10.3) | 14(8.0) | 10(5.7) |
| **P** | 0.744 | 0.744 | 0.317 | 0.515 | <0.001 | 0.417 | 0.262 | 0.731 | 0.555 | 0.944 |
| ***MEIS1*** |  |  |  |  |  |  |  |  |  |  |
| Low (n=182) | 28(15.4) | 17(9.3) | 17(9.3) | 4(2.2) | 94(51.9) | 16(8.8) | 14(7.7) | 17(9.3) | 9(4.9) | 11(6.0) |
| High (n=219) | 73(33.3) | 15(6.8) | 7(3.2) | 89(40.6) | 12(5.5) | 16(8.2) | 40(18.3) | 21(9.6) | 26(11.9) | 12(5.5) |
| **P** | <0.001 | 0.359 | 0.011 | <0.001 | <0.001 | 0.825 | 0.002 | 0.933 | 0.014 | 0.809 |
| ***SPI1*** |  |  |  |  |  |  |  |  |  |  |
| Low (n=218) | 48(22.0) | 11(5.0) | 15(6.9) | 48(22.0) | 78(36.3) | 20(9.2) | 13(6.0) | 29(13.3) | 22(10.1) | 8(3.7) |
| High (n=183) | 53(29.0) | 21(11.5) | 9(4.9) | 45(24.6) | 28(15.3) | 14(7.7) | 41(22.4) | 9(4.9) | 13(7.1) | 15(8.2) |
| **P** | 0.111 | 0.018 | 0.410 | 0.543 | <0.001 | 0.576 | <0.001 | 0.004 | 0.291 | 0.052 |
| ***ERG*** |  |  |  |  |  |  |  |  |  |  |
| Low (n=214) | 47(22.0) | 19(8.9) | 13(6.1) | 66(30.8) | 53(24.9) | 17(7.9) | 29(13.6) | 26(12.1) | 26(12.1) | 9(4.2) |
| High (n=187) | 54(28.9) | 13(7.0) | 11(5.9) | 27(14.4) | 53(28.6) | 17(9.1) | 25(13.4) | 12(6.4) | 9(4.8) | 14(7.5) |
| **P** | 0.112 | 0.478 | 0.937 | <0.001 | 0.397 | 0.669 | 0.957 | 0.051 | 0.009 | 0.159 |
| ***WT1*(missing=2)** | |  |  |  |  |  |  |  |  |  |
| Low (n=193) | 28(14.5) | 15(7.8) | 16(8.3) | 20(10.4) | 81(42.4) | 16(8.3) | 24(12.4) | 18(9.3) | 14(7.3) | 9(4.7) |
| High (n=206) | 72(35.0) | 17(8.3) | 8(3.9) | 73(35.4) | 24(11.7) | 18(8.7) | 30(14.6) | 20(9.7) | 21(10.2) | 14(6.8) |
| **P** | <0.001 | 0.860 | 0.064 | <0.001 | <0.001 | 0.885 | 0.535 | 0.897 | 0.299 | 0.361 |
| ***GATA2*** |  |  |  |  |  |  |  |  |  |  |
| Low (n=189) | 37(19.6) | 16(8.5) | 16(8.6) | 31(16.4) | 49(26.2) | 16(8.5) | 26(13.8) | 18(9.5) | 22(11.6) | 14(7.4) |
| High (n=212) | 64(30.2) | 16(7.5) | 8(3.8) | 62(29.2) | 57(27.0) | 18(8.5) | 28(13.2) | 20(9.4) | 13(6.1) | 9(4.2) |
| **P** | 0.015 | 0.735 | 0.045 | 0.002 | 0.855 | 0.994 | 0.872 | 0.976 | 0.051 | 0.174 |
| ***BAALC*** |  |  |  |  |  |  |  |  |  |  |
| Low (n=228) | 64(28.1) | 15(6.6) | 11(4.8) | 88(38.6) | 46(20.3) | 16(7.0) | 37(16.2) | 21(9.2) | 25(11.0) | 13(5.7) |
| High (n=173) | 37(21.4) | 17(9.8) | 13(7.6) | 5(2.9) | 60(35.1) | 18(10.5) | 17(9.8) | 17(9.8) | 10(5.8) | 10(5.8) |
| **P** | 0.127 | 0.235 | 0.259 | <0.001 | 0.001 | 0.221 | 0.063 | 0.835 | 0.068 | 0.973 |
