# Supplementary material for: Gene mutational pattern and expression level in 560 acute myeloid leukemia patients and their clinical relevance
Source: J Transl Med. 2017 Aug 22;15:178. doi: 10.1186/s12967-017-1279-4 (PMC5568401; doi:10.1186/s12967-017-1279-4)
Supplement: Supplementary file 7 — Additional file 7: Table S5. Multivariate analysis of intermediate risk group. [file 12967_2017_1279_MOESM7_ESM.docx]

**Table S5.** Multivariate analysis of intermediate risk group

| **Variables** | **CR** | | **OS** | | **DFS** | |
| --- | --- | --- | --- | --- | --- | --- |
|  | **OR(95%CI)** | **P** | **HR(95%CI)** | **P** | **HR(95%CI)** | **P** |
| AGE | 0.966(0.953-0.980) | <0.001 | 1.022(1.013-1.030) | <0.001 | 1.016(1.005-1.028) | 0.006 |
| WBC |  | NS |  | NS |  | NS |
| *FLT3*-ITD/TKD |  | NS | 1.524(1.130-2.055) | 0.006 | 1.848(1.218-2.804) | 0.004 |
| Biallelic *CEBPA* |  | NS | 0.552(0.322-0.945) | 0.030 |  | NS |
| NPM1-mut/DNMT3A-wt | 3.184(1.530-6.626) | 0.002 | 0.541(0.360-0.814) | 0.003 |  | NS |
| *DNMT3A* mutation |  | NS |  | NS |  | NS |
| High *MECOM* | 0.542(0.341-0.861) | 0.010 | 1.605(1.209-2.129) | 0.001 | 1.577(1.093-2.275) | 0.015 |
| High *MESI1* | 0.424(0.259-0.694) | 0.001 | 1.524(1.097-2.118) | 0.012 |  | NS |
| High *SPI1* |  | NS |  | NS |  | NS |
| High *WT1* |  | NS |  | NS |  | NS |
