# Supplementary material for: Gene mutational pattern and expression level in 560 acute myeloid leukemia patients and their clinical relevance
Source: J Transl Med. 2017 Aug 22;15:178. doi: 10.1186/s12967-017-1279-4 (PMC5568401; doi:10.1186/s12967-017-1279-4)
Supplement: Supplementary file 8 — Additional file 8: Table S6. CR rate of different gene mutation and expression groups in intermediate risk group (young AML patients). [file 12967_2017_1279_MOESM8_ESM.docx]

**Table S6.**CR rate of different gene mutation and expression groups in intermediate risk group (young AML patients)

| **Gene mutation** | **CR No(%)** | **Gene expression** | **CR No(%)** |
| --- | --- | --- | --- |
| ***FLT3* ITD/TKD** |  | ***MECOM*** |  |
| Mutated | 51(65.4) | Low | 145(78.4) |
| not mutated | 191(74.0) | High | 87(62.1) |
| **P** | 0.136 | **P** | 0.001 |
| ***NRAS*** |  | ***MEIS1*** |  |
| Mutated | 24(82.8) | Low | 125(76.2) |
| not mutated | 218(71.0) | High | 117(68.0) |
| **P** | 0.178 | **P** | 0.094 |
| ***C-KIT(NA=2)*** |  | ***SPI1*** |  |
| Mutated | 16(76.2) | Low | 143(76.5) |
| not mutated | 225(71.9) | High | 99(66.4) |
| **P** | 0.670 | **P** | 0.042 |
| ***DNMT3A*** |  | ***ERG*** |  |
| Mutated | 25(62.5) | Low | 134(75.7) |
| not mutated | 217(73.3) | High | 108(67.9) |
| **P** | 0.153 | **P** | 0.113 |
| ***IDH1*** |  | ***WT1*** |  |
| Mutated | 20(64.5) | Low | 119(73.5) |
| not mutated | 222(72.8) | High | 123(71.5) |
| **P** | 0.328 | **P** | 0.691 |
| ***IDH2*** |  | ***GATA2*** |  |
| Mutated | 16(66.7) | Low | 111(69.8) |
| not mutated | 226(72.4) | High | 131(74.0) |
| **P** | 0.544 | **P** | 0.392 |
| ***NPM1*/*DNMT3A*** |  | ***BAALC*** |  |
| *NPM1*-mut/*DNMT3A*-wt | 42(91.3) | Low | 143(74.9) |
| Others | 200(69.0) | High | 99(68.3) |
| **P** | 0.002 | **P** | 0.182 |
| ***WT1*** |  |  |  |
| Mutated | 24(77.4) |  |  |
| not mutated | 217(71.4) |  |  |
| **P** | 0.476 |  |  |
| ***CEBPA*** |  |  |  |
| biallelic mutated | 55(84.6) |  |  |
| monoallelic mutated | 18(62.1) |  |  |
| not mutated | 166(69.7) |  |  |
| **P** | 0.028 |  |  |
| ***MLL-PTD*** |  |  |  |
| Mutated | 13(65.0) |  |  |
| not mutated | 229(72.5) |  |  |
| **P** | 0.471 |  |  |
