# Supplementary material for: Gene mutational pattern and expression level in 560 acute myeloid leukemia patients and their clinical relevance
Source: J Transl Med. 2017 Aug 22;15:178. doi: 10.1186/s12967-017-1279-4 (PMC5568401; doi:10.1186/s12967-017-1279-4)
Supplement: Supplementary file 9 — Additional file 9: Table S7. Multivariate analysis of prognostic value of young AML patients. [file 12967_2017_1279_MOESM9_ESM.docx]

**Table S7.** Multivariate analysis of prognostic value of young AML patients

| **Variables** | **CR** | | **OS** | | **DFS** | |
| --- | --- | --- | --- | --- | --- | --- |
|  | **OR(95%CI)** | **P** | **HR(95%CI)** | **P** | **HR(95%CI)** | **P** |
| Age | 0.973(0.959-0.987) | <0.001 | 1.017(1.009-1.026) | <0.001 | 1.015(1.003-1.026) | 0.012 |
| WBC |  | NS |  | NS |  | NS |
| Cytogenetic risk |  | NS | 1.089(1.022-1.162) | 0.009 | 1.106(1.017-1.202) | 0.018 |
| *FLT3*-ITD/TKD |  | NS | 1.635(1.204-2.220) | 0.002 | 1.611(1.058-2.452) | 0.026 |
| Biallelic *CEBPA* |  | NS | 0.433(0.264-0.713) | 0.001 |  | NS |
| NPM1-mut/DNMT3A-wt | 7.297(2.469-21.565) | <0.001 | 0.516(0.322-0.825) | 0.006 |  | NS |
| *MLL*-PTD |  | NS |  | NS |  | NS |
| *DNMT3A* mutation |  | NS |  | NS |  | NS |
| High *MECOM* | 0.539(0.347-0.838) | 0.006 | 1.664(1.272-2.177) | <0.001 | 1.808(1.307-2.501) | <0.001 |
| High *MESI1* | 0.440(0.282-0.686) | <0.001 |  | NS |  | NS |
| High *SPI1* |  | NS |  | NS |  | NS |

NS, no significance
