# Supplementary material for: Gene mutational pattern and expression level in 560 acute myeloid leukemia patients and their clinical relevance
Source: J Transl Med. 2017 Aug 22;15:178. doi: 10.1186/s12967-017-1279-4 (PMC5568401; doi:10.1186/s12967-017-1279-4)
Supplement: Supplementary file 10 — Additional file 10: Table S8. Multivariate analysis of intermediate risk group (young AML patients). [file 12967_2017_1279_MOESM10_ESM.docx]

**Table S8.** Multivariate analysis of intermediate risk group (young AML patients)

| **Variables** | **CR** | | **OS** | | **DFS** | |
| --- | --- | --- | --- | --- | --- | --- |
|  | **OR(95%CI)** | **P** | **HR(95%CI)** | **P** | **HR(95%CI)** | **P** |
| AGE | 0.969(0.951-0.987) | 0.001 | 1.018(1.007-1.029) | 0.001 | 1.016(1.001-1.030) | 0.033 |
| WBC |  | NS |  | NS |  | NS |
| *FLT3*-ITD/TKD |  | NS | 1.787(1.273-2.509) | 0.001 | 2.018(1.287-3.164) | 0.002 |
| Biallelic *CEBPA* |  | NS | 0.412(0.240-0.708) | 0.001 |  | NS |
| NPM1-mut/DNMT3A-wt | 6.212(2.047-18.849) | 0.001 | 0.517(0.312-0.856) | 0.010 |  | NS |
| *DNMT3A* mutation |  | NS |  | NS |  | NS |
| High *MECOM* | 0.520(0.307-0.881) | 0.015 | 1.579(1.140-2.186) | 0.006 | 1.578(1.062-2.342) | 0.024 |
| High *MESI1* | 0.491(0.287-0.843) | 0.010 |  | NS |  | NS |
| High *SPI1* |  | NS |  | NS |  | NS |
| High *WT1* |  | NS |  | NS |  | NS |

NS, no significance
