# Supplementary material for: Gene mutational pattern and expression level in 560 acute myeloid leukemia patients and their clinical relevance
Source: J Transl Med. 2017 Aug 22;15:178. doi: 10.1186/s12967-017-1279-4 (PMC5568401; doi:10.1186/s12967-017-1279-4)
Supplement: Supplementary file 11 — Additional file 11: Figure S3. Kaplan–Meier curves for OS and DFS according to genotypes. [file 12967_2017_1279_MOESM11_ESM.docx]

Figure S3. Kaplan-Meier curves for OS and DFS according to genotypes. (A-B) The median OS and DFS of patients with biallelic or mono allelic CEBPA mutations were not reached (NR) and 12±3.9 months (P<0.001), and NR and 21±6.5 months (P=0.002), compared with wild-type CEBPA patients. The hazard ratios (HR) of biallelic *CEBPA* mutation were depicted. (C-D) The median OS and DFS of patients with or without FLT3-ITD/TKD mutations were 9±0.9 months versus 25±3.9 months (P<0.001), and 9±2.2 months versus 33±6.1 months (P<0.001), respectively. (E-F) The median OS and DFS of patients with or without DNMT3A mutation were 11±1.5 months versus 20±2.9 months (P =0.014), and 12±2.6 months versus 30±6.6 months (P =0.023), respectively. (G-H) The median OS and DFS of patients subgroup that carried NPM1 mutation with or without FLT3-ITD/TKD mutations were 9±3.2 months versus 30±7.8 months (P =0.065) and 9±2.0 months versus 33±6.5 months (P =0.080), respectively. (I-J) The median OS and DFS of patients subgroup that carried NPM1 mutation with or without DNMT3A mutation were 9±3.2 months versus 30±9.2 months (P =0.010) and 11±1.4 months versus 36±12.9 months (P =0.044), respectively.


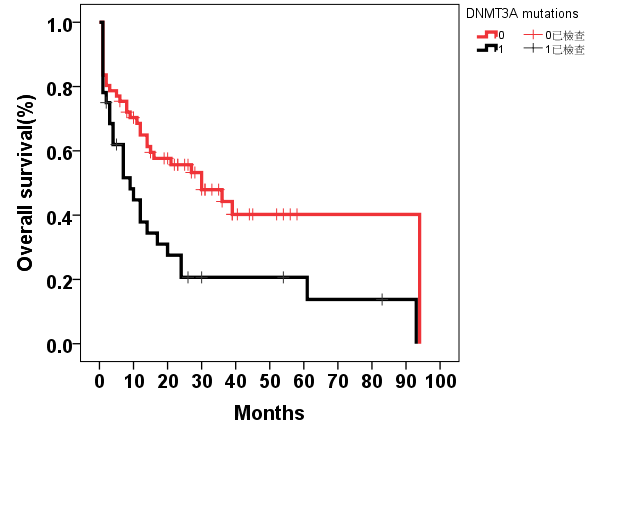

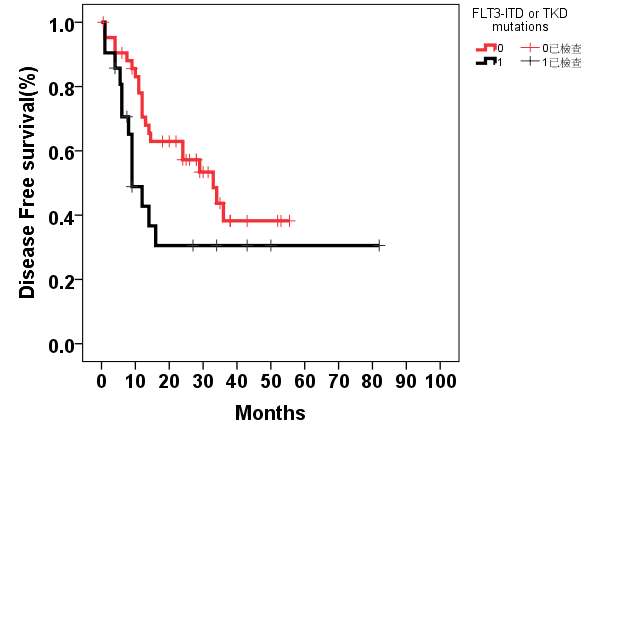

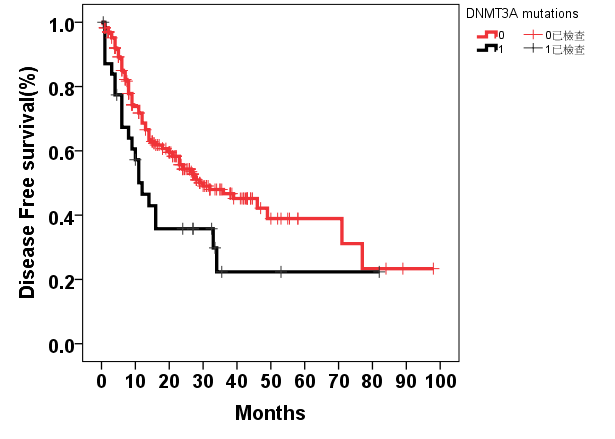

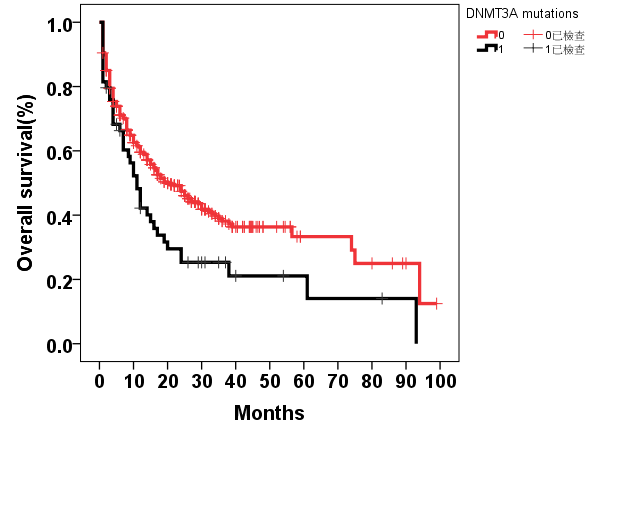

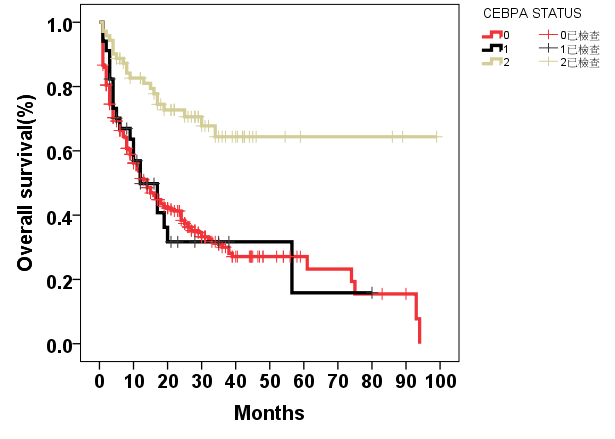

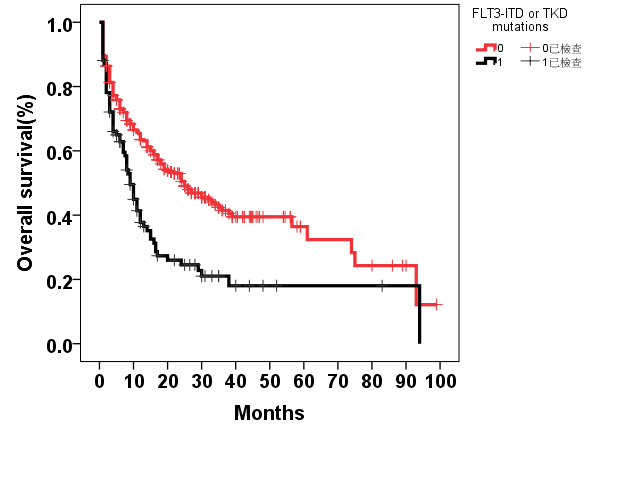

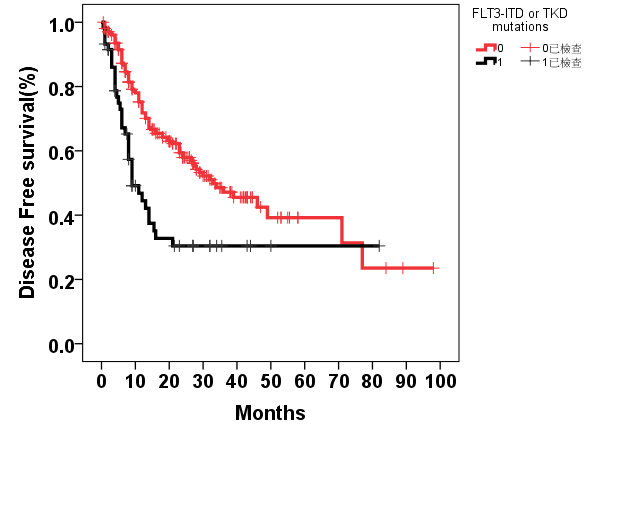

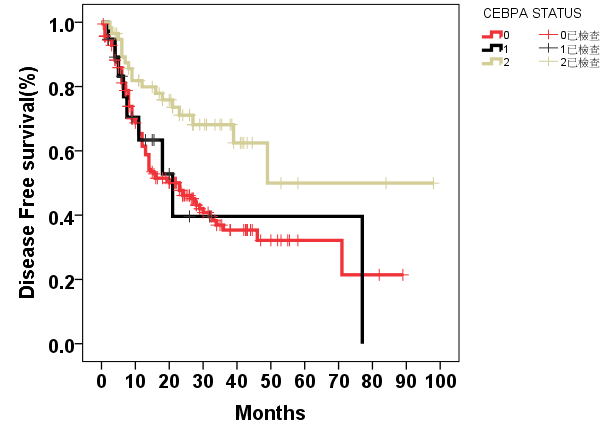
**A B C D**

**CEBPA biallelic(n=71)**

**CEBPA biallelic(n=57)**

**FLT3 -ITD/TKDwt(n=300)**

**FLT3 -ITD/TKDwt(n=205)**

**CEBPA monoallelic(n=34)**

**CEBPA monoallelic(n=19)**

**FLT3 -ITD/TKDmut(n=101)**

**CEBPA wt(n=185)**

**CEBPA wt(n=292**)

**FLT3 -ITD/TKDmut(n=59)**

**HR= 2.047(95%CI:1.382-3.301)**

**P<0.001**

**HR =0.691(95%CI:0.544-0.878)**

**P=0.002**

**HR=1.807(95%CI:1.336-2.391)**

**P<0.001**

**HR =0.329(95%CI:0.209-0.517)**

**P<0.001**


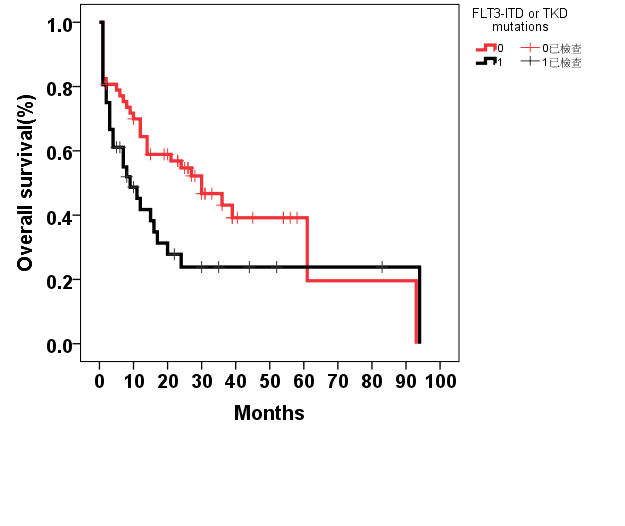
**E F G H**

**NPM1mut/FLT3-ITDwt(n=43)**

**DNMT3Awt(n=232)**

**NPM1mut/FLT3-ITDwt(n=57)**

**DNMT3Awt(n=347)**

**NPM1mut/FLT3-ITDmut(n=21)**

**DNMT3Amut(n=54)**

**DNMT3Amut(n=32)**

**NPM1mut/FLT3-ITDmut(n=36)**

**HR=1.865(95%CI:0.928-3.748)**

**P=0.080**

**HR=1.648(95%CI:0.970-2.800)**

**P=0.065**

**HR=1.724(95%CI:1.078-2.757)**

**P=0.023**

**HR=1.532(95%CI:1.092-2.149)**

**P=0.014**


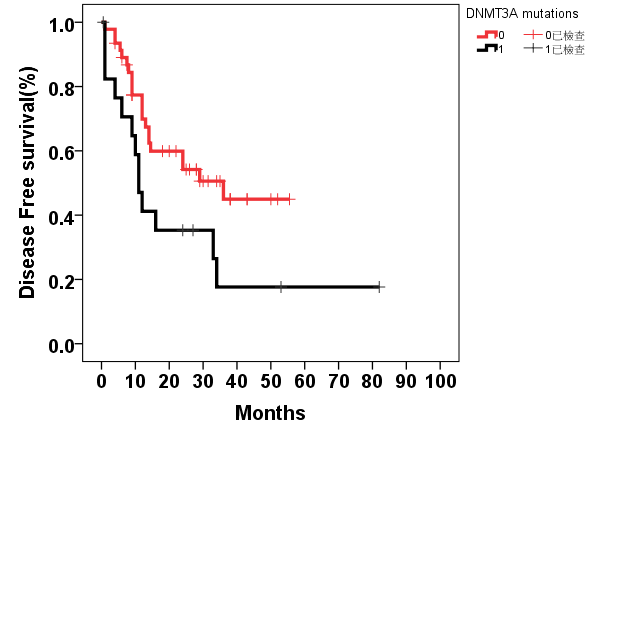
**I J**

**NPM1mut/DNMT3Awt(n=46)**

**NPM1mut/DNMT3Awt(n=61)**

**NPM1mut/DNMT3Amut(n=18)**

**NPM1mut/DNMT3Amut(n=32)**

**HR=2.039(95%CI:1.020-4.078)**

**P=0.044**

**HR=2.009(95%CI:1.184-3.409)**

**P=0.010**
