# Supplementary material for: Gene mutational pattern and expression level in 560 acute myeloid leukemia patients and their clinical relevance
Source: J Transl Med. 2017 Aug 22;15:178. doi: 10.1186/s12967-017-1279-4 (PMC5568401; doi:10.1186/s12967-017-1279-4)
Supplement: Supplementary file 12 — Additional file 12: Figure S4. Kaplan–Meier curves of young AML patients for OS and DFS according to genotypes. [file 12967_2017_1279_MOESM12_ESM.docx]

Figure S4. Kaplan-Meier curves of young AML patients for OS and DFS according to genotypes. (A-B) The median OS and DFS of patients with biallelic or mono allelic CEBPA mutations were not reached (NR) and 12±3.4 months (P<0.001), and NR and 21±7.7 months (P=0.004), compared with wild-type CEBPA patients. The hazard ratios (HR) of biallelic *CEBPA* mutation were depicted. (C-D) The median OS and DFS of patients with or without FLT3-ITD/TKD mutations were 10±1.2 months versus 32±4.3 months (P<0.001), and 9±2.4 months versus 36±6.7 months (P<0.001), respectively. (E-F) The median OS and DFS of patients with or without DNMT3A mutation were 11±1.8 months versus 27±3.8 months (P =0.006), and 10±2.4 months versus 36±7.0 months (P =0.007), respectively. (G-H) The median OS and DFS of patients subgroup that carried NPM1 mutation with or without FLT3-ITD/TKD mutations were 11±3.9 months versus 39±8.4 months (P =0.003) and 9±2.6 months versus 34±4.1 months (P =0.014), respectively. (I-J) The median OS and DFS of patients subgroup that carried NPM1 mutation with or without DNMT3A mutation were 10±3.8 months versus 39 months (P =0.002) and 11±1.9 months versus 36 months (P =0.029), respectively.


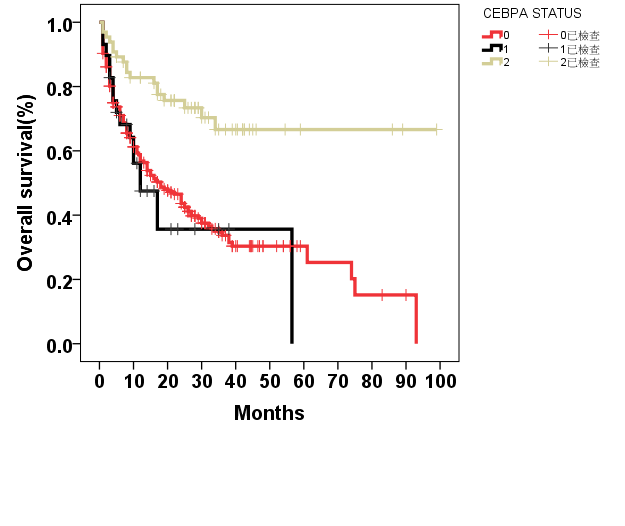

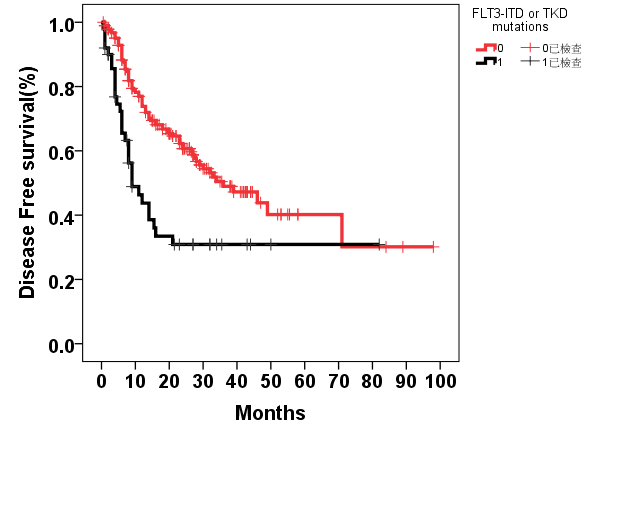

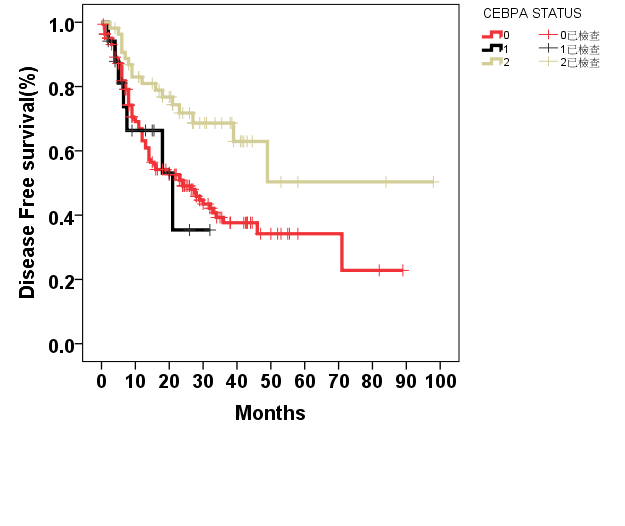

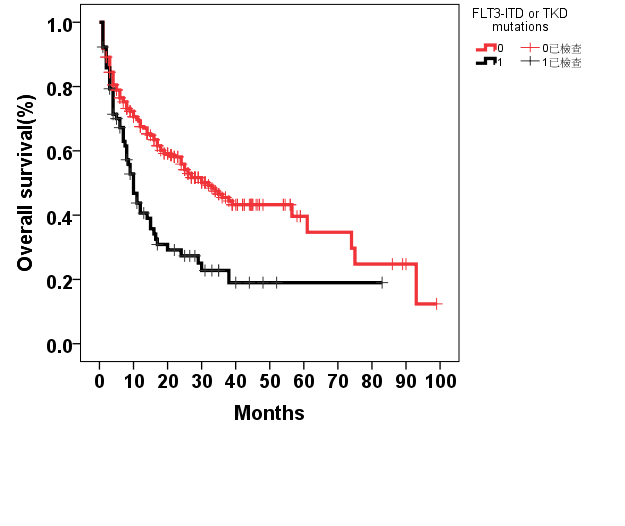
**A B C D**

**CEBPA biallelic(n=65)**

**CEBPA biallelic(n=54)**

**FLT3 -ITD/TKDwt(n=258)**

**FLT3 -ITD/TKDwt(n=187)**

**CEBPA monoallelic(n=29)**

**CEBPA monoallelic(n=17)**

**FLT3 -ITD/TKDmut(n=78)**

**CEBPA wt(n=163)**

**CEBPA wt(n=238**)

**FLT3 -ITD/TKDmut(n=50)**

**HR =0.468(95%CI:0.278-0.790)**

**P=0.004**

**HR =0.337(95%CI:0.206-0.551)**

**P<0.001**

**HR= 2.168(95%CI:1.420-3.310)**

**P<0.001**

**HR=1.911(95%CI:1.385-2.637)**

**P<0.001**


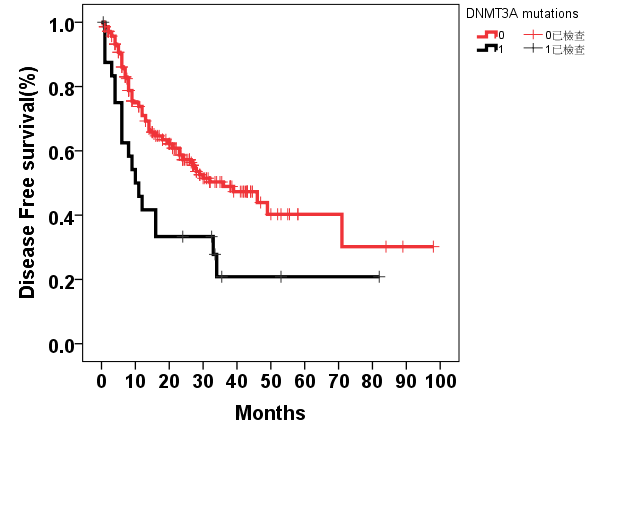

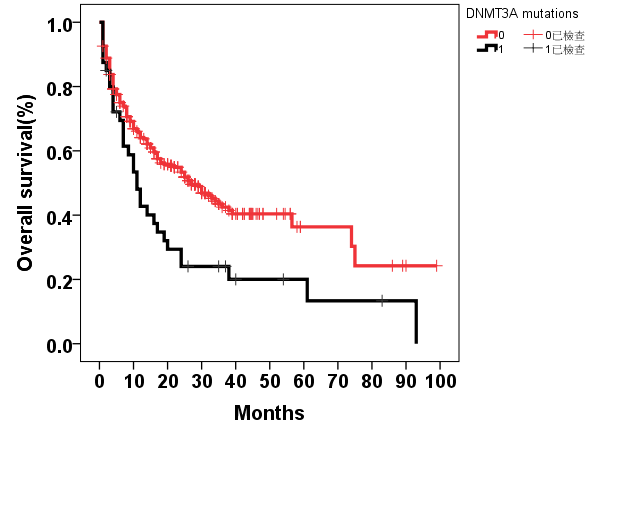

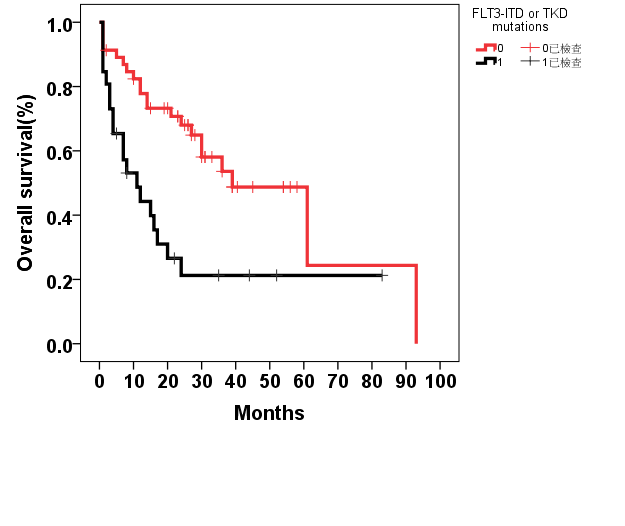
**E F G H**


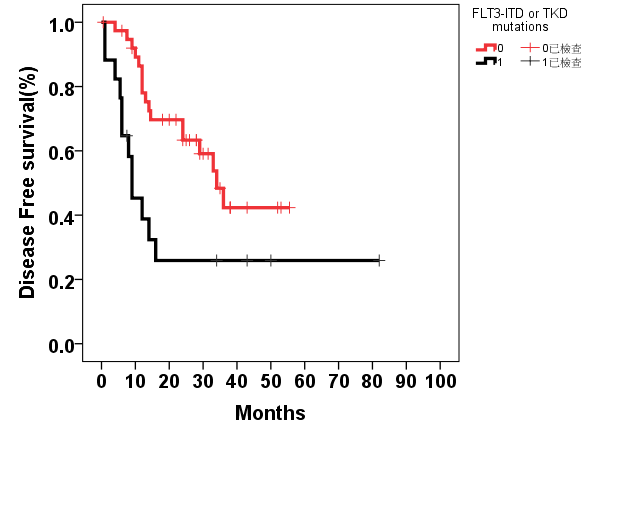


**DNMT3Awt(n=212)**

**NPM1mut/FLT3-ITDwt(n=46)**

**DNMT3Awt(n=296)**

**NPM1mut/FLT3-ITDwt(n=39)**

**DNMT3Amut(n=25)**

**NPM1mut/FLT3-ITDmut(n=17)**

**DNMT3Amut(n=40)**

**NPM1mut/FLT3-ITDmut(n=26)**

**HR=2.014(95%CI:1.213-3.346)**

**P=0.007**

**HR=1.716(95%CI:1.167-2.524)**

**P=0.006**

**HR=2.557(95%CI:1.213-5.393)**

**P=0.014**

**HR=2.669(95%CI:1.410-5.053)**

**P=0.003**

**I J**


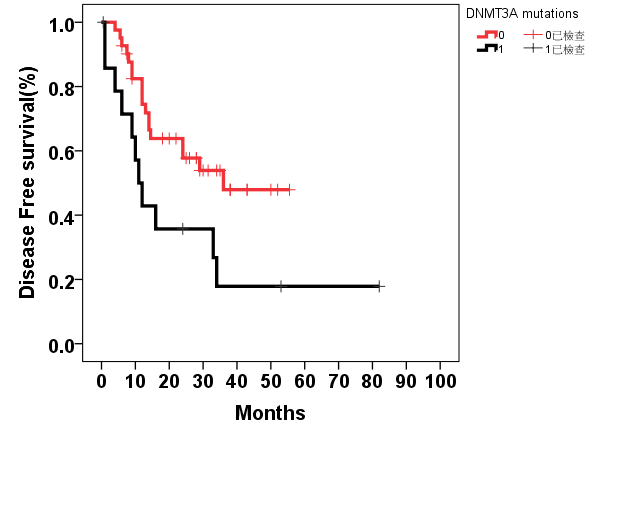

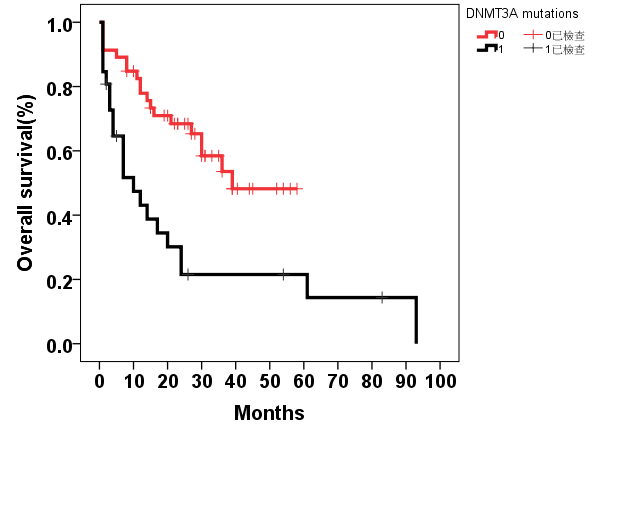


**NPM1mut/DNMT3Awt(n=41)**

**NPM1mut/DNMT3Awt(n=46)**

**NPM1mut/DNMT3Amut(n=26)**

**NPM1mut/DNMT3Amut(n=15)**

**HR=2.306(95%CI:1.087-4.890)**

**P=0.029**

**HR=2.785(95%CI:1.463-5.301)**

**P=0.002**
