# Supplementary material for: Gene mutational pattern and expression level in 560 acute myeloid leukemia patients and their clinical relevance
Source: J Transl Med. 2017 Aug 22;15:178. doi: 10.1186/s12967-017-1279-4 (PMC5568401; doi:10.1186/s12967-017-1279-4)
Supplement: Supplementary file 13 — Additional file 13: Figure S5. Comparison of OS and DFS between young AML patients with low or high gene expression with statistical significance in univariate analysis. [file 12967_2017_1279_MOESM13_ESM.docx]

Figure S5. Comparison of OS and DFS between young AML patients with low or high gene expression with statistical significance in univariate analysis. (A-B) The median OS and DFS of patients with low or high *MECOM* expression were 34±8.0 months versus13±1.9 months (P <0.001), and 39±8.5 months versus 20±7.6 months (P =0.033), respectively. (C-D) The median OS and DFS of patients with low or high *MEIS1* expression were 35±9.1 months versus 15±2.7 months (P=0.002*)*, and 39±8.2 months versus 23±5.5 months (P =0.039), respectively. (E-F) The median OS and DFS of patients with low or high *SPI1* expression were 33±5.0 months versus 16±2.6 months (P =0.009), and 39±8.0 months versus 23±6.4 months (P =0.043), respectively. (G-H) The median OS and DFS of patients with low or high *WT1* expression were 30±6.4 months versus 19±4.2 months (P=0.172) and 39±13.6 months versus 24±5.6 months (P=0.069), respectively. (I-J) Patients with low expression of both *MECOM* and *MEIS1* were compared to those with either or both high expression of *MECOM* and *MEIS1*. The median OS were 56.5±17.2 months, 18±4.4 months and 12±2.7 months (P<0.001). The median DFS were 46±11.6 months, 33±6.1 months and 13±2.7 months (P=0.034). The hazard ratios (HR) of High *MECOM* or *MEIS1* for OS and DFS were 1.976 (95%CI: 1.345-2.903) and 1.270(95%CI: 0.799-2.017), respectively, while the HR of High *MECOM* and High *MEIS1* were 2.636 (95%CI: 1.740-3.992) and 1.928(95%CI: 1.170-3.176), respectively. (K-L) The OS and DFS were compared in a subgroup of patients with low expression of both *MECOM* and *MEIS1.* The median OS and DFS of patients with low or high *WT1* expression were 56.5 months versus 19±7.1 months (P =0.004), and NR versus 19.5±5.8 months (P =0.015), respectively.

A B C D


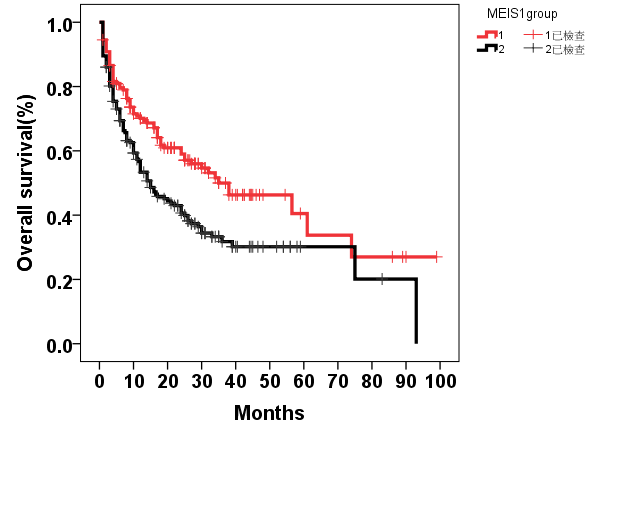

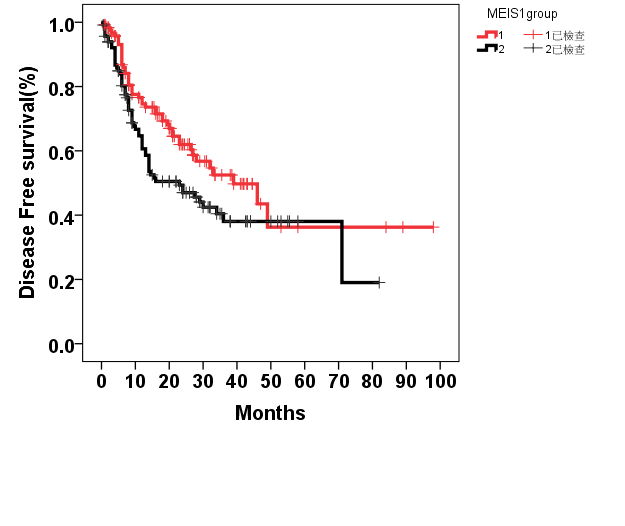

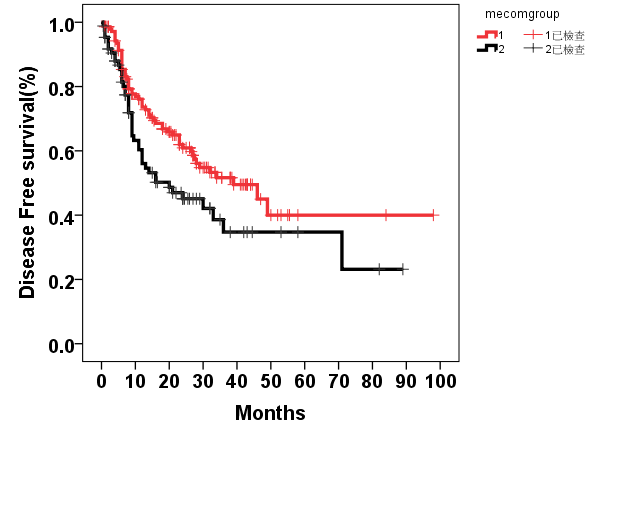

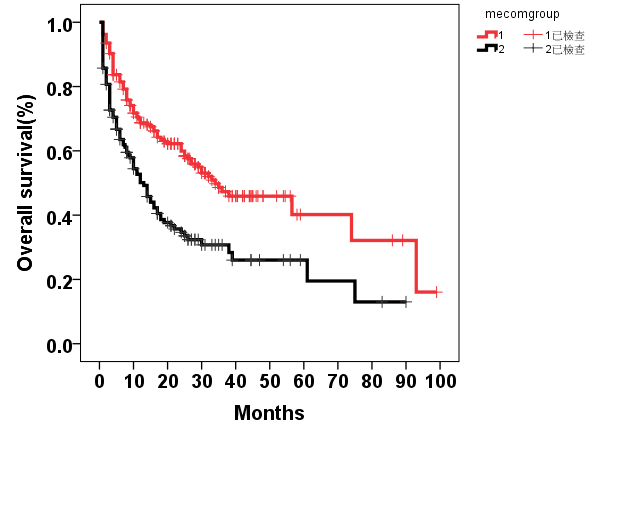


**Low MESI1(n=121)**

**Low MESI1(n=164)**

**Low MECOM(n=141)**

**Low MECOM(n=185)**

**High MECOM(n=140)**

**High MECOM(n=86)**

**High MESI1(n=116)**

**High MESI1(n=172)**

**HR=1.492(95%CI:1.020-2.184)**

**P=0.039**

**HR=1.533(95%CI:1.036-2.269)**

**P=0.033**

**HR=1.893 (95%CI:1.405-2.551)**

**P<0.001**

**HR=1.605(95%CI:1.191-2.162)**

**P=0.002**

E F G H


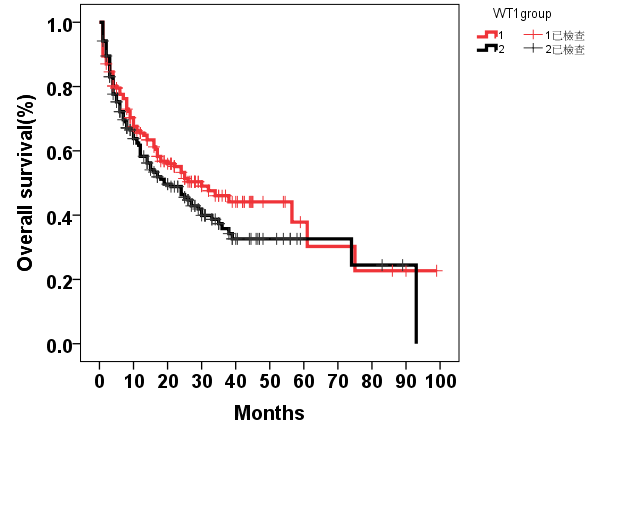

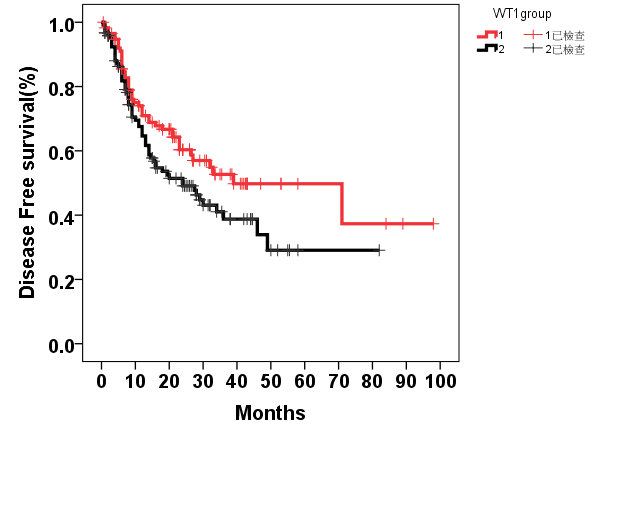

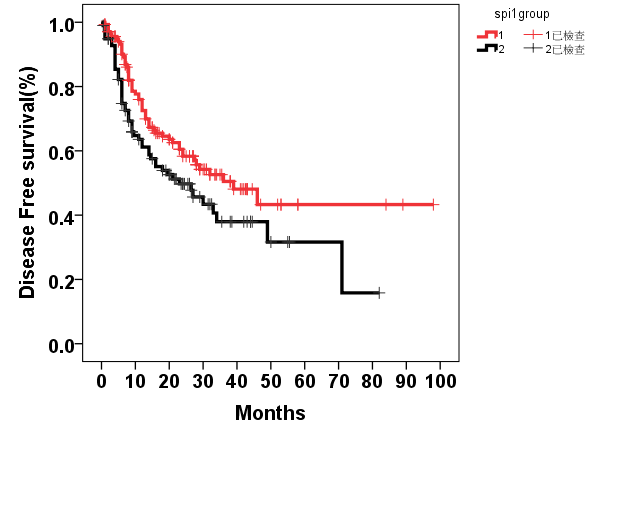

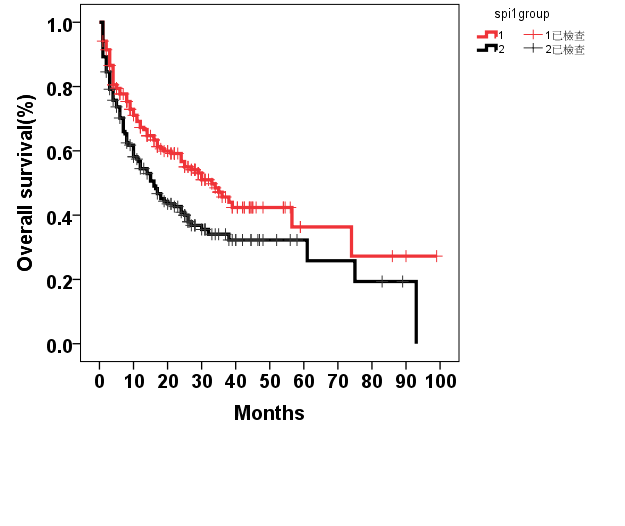


**Low WT1(n=162)**

**Low WT1(n=116)**

**Low SPI1(n=139)**

**Low SPI1(n=187)**

**High SPI1(n=149)**

**High WT1(n=172)**

**High WT1(n=121)**

**High SPI1(n=98)**

**HR=1.475(95%CI:1.101-1.977)**

**P=0.009**

**HR=1.229(95%CI:0.914-1.651)**

**P=0.172**

**HR=1.476(95%CI:1.012-2.154)**

**P=0.043**

**HR=1.425(95%CI:0.972-2.089)**

**P=0.069**

I J K L

**Low MECOM and Low MEIS1**

**Low MECOM and Low MEIS1**


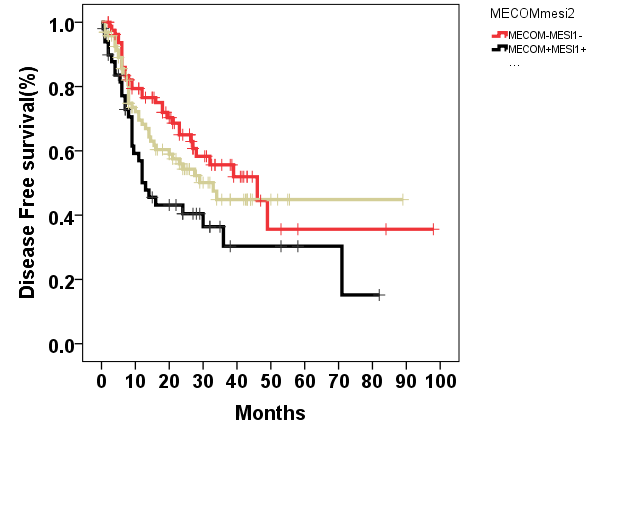

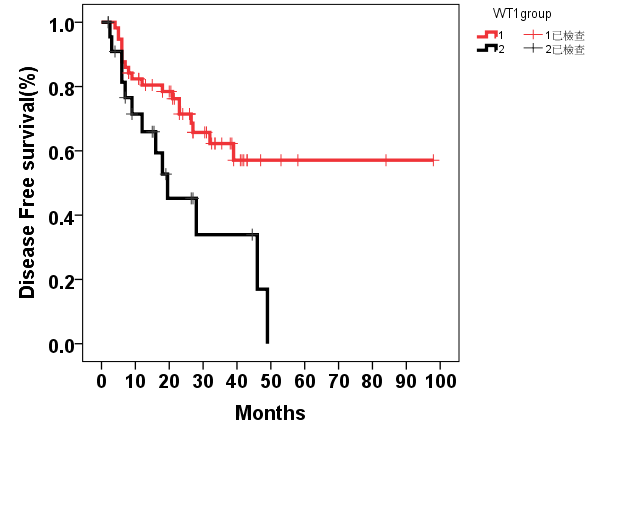

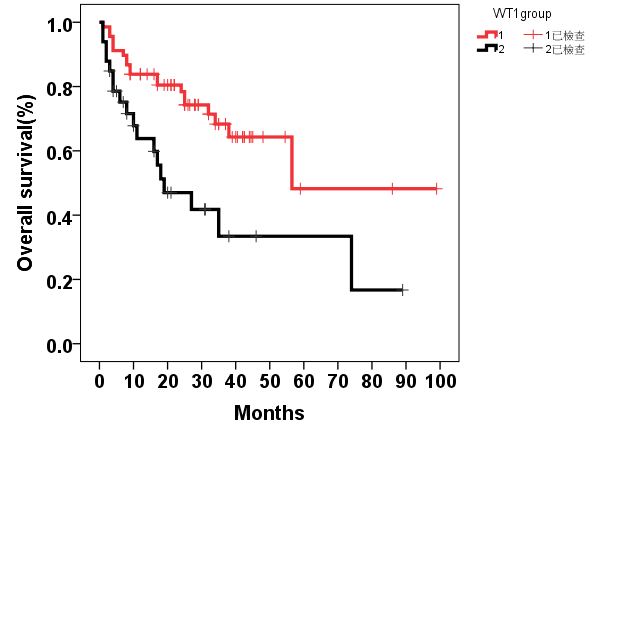

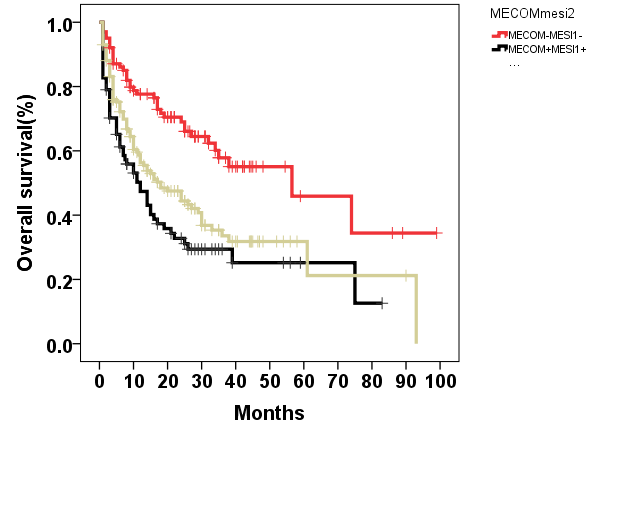


**Low WT1(n=68)**

**Low WT1(n=57)**

**Low MECOM, Low MEIS1(n=81)**

**Low MECOM and Low MEIS1(n=101)**

**High MECOM or High MEIS1(n=96)**

**High WT1(n=24)**

**High WT1(n=33)**

**High MECOM or High MEIS1(n=143)**

**High MECOM and High MEIS1 (n=50)**

**High MECOM and High MEIS1 (n=81)**

**HR=2.599(95%CI: 1.365-4.951)**

**P=0.004**

**HR=2.425(95%CI: 1.191-4.940)**

**P=0.015**

**P<0.001**

**P=0.034**
