# Supplementary material for: Gene mutational pattern and expression level in 560 acute myeloid leukemia patients and their clinical relevance
Source: J Transl Med. 2017 Aug 22;15:178. doi: 10.1186/s12967-017-1279-4 (PMC5568401; doi:10.1186/s12967-017-1279-4)

Figure S6. Risk stratification of young AML according to gene mutations and gene expression level. Low risk: biallelic *CEBPA* mutation; Intermediate risk I: low *MECOM* and *MEIS1* without biallelic *CEBPA* mutation or *NPM1*-mut/*DNMT3A*-wt; Intermediate risk II: others; High risk: *FLT3*-ITD/TKD with the absence of *NPM1*-mut/*DNMT3A*-wt or *DNMT3A* mutation or high *MECOM* and *MEIS1*. (A) OS, (B) DFS.

A B


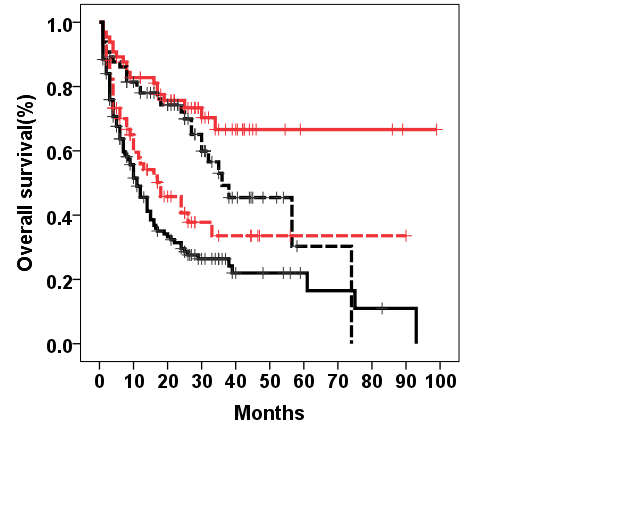


**Low risk:**

median DFS: NR (n=54)

**Low risk:**

median OS: NR (n=65)

**Intermediate risk I:**

median DFS: 32±8.7mo (n=54)

HR=1.479(95%CI: 0.789-2.770)

**Intermediate riskI:**

median OS: 36±7.5mo (n=65)

HR=1.672(95%CI: 0.924-3.026)

**Intermediate risk II:**

median DFS: 27.5mo (n=44)

HR=1.709(95%CI: 0.872-3.351)

**Intermediate riskII:**

median OS: 18±5.2mo (n=68)

HR=2.909(95%CI: 1.652-5.120)

**High risk：**

median DFS:12±1.6mo(n=85)

HR=2.977(95%CI: 1.717-5.164)

**High risk：**

median OS: 11±1.5mo(n=138)

HR=3.956(95%CI: 2.387-6.555)

P<0.001

P<0.001


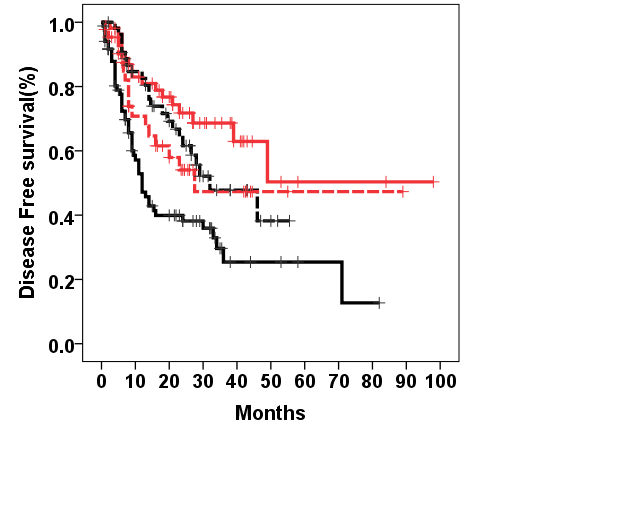

Supplement: Supplementary file 14 — Additional file 14: Figure S6. Risk stratification of young AML according to gene mutations and gene expression levels. [file 12967_2017_1279_MOESM14_ESM.docx]
